# Critical Investigation of Betaine Hydrochloride‐Based Deep Eutectic Solvent for Ionometallurgical Metal Production

**DOI:** 10.1002/open.202300114

**Published:** 2023-08-07

**Authors:** Janine Richter, Tobias Pietsch, Noah Elsner, Michael Ruck

**Affiliations:** ^1^ Faculty of Chemistry and Food Chemistry Technische Universität Dresden 01062 Dresden Germany; ^2^ Max Planck Institute for Chemical Physics of Solids Nöthnitzer Str. 40 01187 Dresden Germany

**Keywords:** deep eutectic solvents, electrodeposition, green solvents, ionic liquids, metal oxides

## Abstract

The applicability of a deep eutectic solvent (DES) consisting of betainium hydrochloride, urea and glycerol is examined with respect to ionometallurgical metal extraction and compared with the ionic liquid (IL) betainium bis(trifluoromethylsulfonyl)imide ([Hbet][NTf_2_]). The DES dissolves numerous metal oxides, where not only betaine and chloride act as stabilizing ligands, but also nascent ammonia seems to be essential. From such solutions, cobalt, copper, zinc, tin, lead, and even vanadium can be electrodeposited, demonstrating the feasibility of ionometallurgy. However, repeated recycling of the DES is not conceivable. NMR spectroscopy and mass spectrometry identify numerous decomposition reactions taking place at 60 °C already. The by‐products that are formed not only make recycling more difficult, but also pose a toxicity problem. The opportunities and obstacles of DESs and ILs for their application in ionometallurgy are critically discussed. It is shown that a thorough understanding of the underlying chemical processes is critical.

## Graphical abstract

The applicability of a deep eutectic solvent (DES), formed by betainium hydrochloride, urea and glycerol, for ionometallurgical metal production is investigated and critically discussed. Although several metals can be electrodeposited after rapid dissolution of the respective metal oxide, various decomposition reactions prevent the solvent from being usable and environmentally friendly.

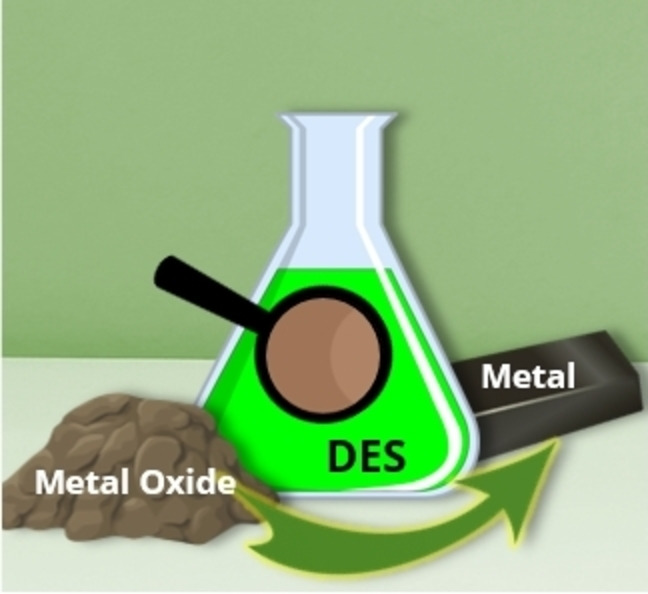

## Introduction

Hundreds of millions of tons of metals are needed every year. Metal production typically involves three main steps: activating, separating and reducing. Activation of ores, earths, minerals or scrap involves either high temperatures or aggressive media. While pyrometallurgy is extremely energy‐intensive and CO_2_‐emitting, hydrometallurgical processing requires harmful chemicals, a lot of water, and generates large volumes of waste water. Also electrochemical metal production in conventional molten salt electrolysis, with the Hall‐Héroult process (electrodeposition of aluminum at approximately 1000 °C) as the industrially most relevant example, involves immense energy consumption and environmentally problematic electrolytes.^[1]^ Remarkably, the underlying processes are usually a hundred or more years old. Because they are highly optimized and provide vast quantities of metals, disruptive innovation in this area is a thing of the past. It is time to explore whether we can use 21^st^ century knowledge and capabilities to create much more sustainable, yet profitable, alternatives.

One of these innovative approaches is called ionometallurgy,[^2^, ^3^, ^4^] which is the production of metals and valuable metal compounds using ionic liquids (ILs)[^4^, ^5^, ^6^, ^7^, ^8^, ^9^, ^10^] or deep eutectic solvents (DESs)[^11^, ^12^, ^13^, ^14^, ^15^] as reaction media. Both can dissolve, usually under moderate heating, many metal oxides and sulfides,[^16^, ^17^, ^18^] that is, compounds that occur in, or are very similar to, common natural metal resources. ILs and DESs have also been employed in metal separation processes, and direct electrodeposition of metals from such solutions has been demonstrated.[^2^, ^4^, ^9^, ^10^, ^19^, ^20^]

Although ILs and DESs are often considered as similar classes of solvents, different interactions are effective on the molecular level, having significant implications for their properties. ILs are salts with a melting point below 100 °C, that is, they consist of defined cations and anions interacting electrostatically with each other. Among their favorable properties are negligible volatility, low flammability, high conductivity and high thermal and chemical stability.^[21]^ In contrast, in DESs, at least one hydrogen bond donor and one acceptor form a mixture whose melting point is below the melting points of the individual components. Thus, interactions between the uncharged molecules in a DES are primarily based on a hydrogen bond network.[^22^, ^23^]

An advantage of DESs over ILs is their convenient preparation by simply mixing cheap and nontoxic reagents that are often found in nature or can be easily obtained from natural products. In contrast, most ILs require complex synthesis, which not only makes them more expensive, but often less environmentally friendly. In fact, the situation is much more complex and requires a differentiated and in‐depth analysis of strengths and weaknesses of ILs and DESs.[^23^, ^24^]

Among the common disadvantages of DESs are significantly higher vapor pressures and lower thermal stability than in the case of ILs. Although the vapor pressure of a typical DES lies several magnitudes below those of ordinary organic single‐compound solvents, it is not negligible, which especially becomes relevant at elevated temperatures. For a DES consisting of components with different volatility, this can involve divergent evaporation, changing the composition of the DES.[^24^, ^25^]

The reason for the often overestimated thermal stability of DESs is seen in insufficient or inappropriate methods of analysis. For example, thermogravimetric measurements, performed by heating DESs with a small heating rate, are not suitable for estimating their long‐term thermal stability. As has also been demonstrated for ILs,[^26^, ^27^] their long‐term is often significantly lower than their short‐term stability.[^24^, ^25^, ^28^, ^29^] High‐temperature reactions involve the decomposition of DES components, forming toxic by‐products.[^13^, ^30^] Furthermore, the decomposition of urea was shown to be enhanced by polyalcohols, common components of a DES, at temperatures as low as 80 °C, which is significantly below the decomposition temperature of pure urea (133 °C).[^31^, ^32^]

Also the chemical stability of DESs has to be questioned, as DESs containing a carboxylic acid and an alcohol were reported to undergo esterification up to 35 % even at room temperature.^[13]^ When it comes to electrochemistry, several DESs were shown to decompose under long‐term application of an electrical potential, whereas numerous toxic and even chlorinated by‐products form.[^33^, ^34^] Such by‐products not only pose a problem regarding the recycling of the DES, but they must also be taken into account as additional components that change the solvent properties. Moreover, the occurrence of poorly characterized by‐products raises serious doubt about the non‐toxicity and biocompatibility of DESs. Therefore DESs, like ILs,[^24^, ^35^] cannot generally be categorized as green solvents.

For use of an IL or a DES as solvent for metal oxides, that is, in the first step of ionometallurgical processing, the presence of Brønsted‐acidic functional groups, for example in carboxylic acids, has proven advantageous.[^5^, ^16^, ^36^, ^37^, ^38^, ^39^] While the protons are consumed to form water in an acid‐base reaction, which is then thermally evaporated, the carboxylate groups stabilize the metal cations in coordination compounds.

Among ILs, betainium bis(trifluoromethylsulfonyl)imide, [Hbet][NTf_2_], is especially suited[^36^, ^40^] as demonstrated for the ionometallurgical production of lead,[^9^, ^41^, ^42^] plutonium,^[20]^ copper,[^4^, ^42^] zinc[^9^, ^42^] and cobalt^[10]^ from their respective oxides. In all cases, this means a considerable reduction in energy consumption and a more efficient process, at least from a chemical point of view. However, also the environmental impact of the IL, its production and its recycling have to be taken into account for sustainable processes. Moreover, to maintain the chance of replacing established production processes, an ionometallurgical approach must also be economically attractive. Regarding [Hbet][NTf_2_], betaine is a naturally occurring by‐product from sugar production,^[43]^ therefore highly available, non‐toxic and biodegradable. In contrast, the [NTf_2_]^−^ anion must be expensively synthesized and is weakly toxic,^[44]^ impeding large‐scale commercial application. Unfortunately, other betainium salts feature significantly higher melting points which increases processing temperatures.^[36]^

A low‐cost alternative could be a DES that includes betainium. Kuperkar et al. presented a series of such DESs, consisting of betainium hydrochloride ([Hbet]Cl) as hydrogen bond acceptor (HBA), urea (U) as a primary hydrogen bond donor (HBD) and ethylene glycol (EG), diethylene glycol (DEG), triethylene glycol (TEG), or glycerol (GLY) as secondary HBD.^[45]^ Especially the mixture of [Hbet]Cl, U and GLY in the molar ratio of 1 : 4 : 2.5 ([Hbet]Cl/4U/2.5GLY) with a reported thermal stability of up to 194 °C^[45]^ seemed promising for testing in ionometallurgical processes.

We compared the solubility of 30 metal oxides in the DES [Hbet]Cl/4U/2.5GLY at 150 °C to those using the IL [Hbet]_2_[NTf_2_]Cl at 175 °C.^[24]^ Then, we tested the electrodeposition of V, Mn, Co, Cu, Zn, Mo, Sn and Pb from such DES solutions. Experimental observations stimulated an investigation of the chemical and thermal stability of the used DES by NMR spectroscopy and mass spectrometry. Based on the performance of these betainium‐based solvents, we critically discuss fundamental aspects of the applicability of ILs and DESs with respect to synthesis, stability and recyclability.

## Results and Discussion

### Properties and stability of [Hbet]Cl/4U/2.5GLY

The previously reported thermal stability of [Hbet]Cl/4U/2.5GLY up to 194 °C^[45]^ was questioned by the experimental observation of an ammonia odor and an increasing yellow coloration when the DES was heated even well below its nominal decomposition temperature. Also contrary to the previous report,^[45]^ we observed that the DES does not form a stable liquid at room temperature, but starts to crystallize within a few hours. By storing it at 60 °C, the DES remained liquid, but a yellow color was observed after several days, suggesting decomposition reactions. Therefore, the DES was studied by ^1^H and ^13^C NMR spectroscopy as well as mass spectrometry directly after synthesis and after 33 days storage at 60 °C.

**Figure 1:**
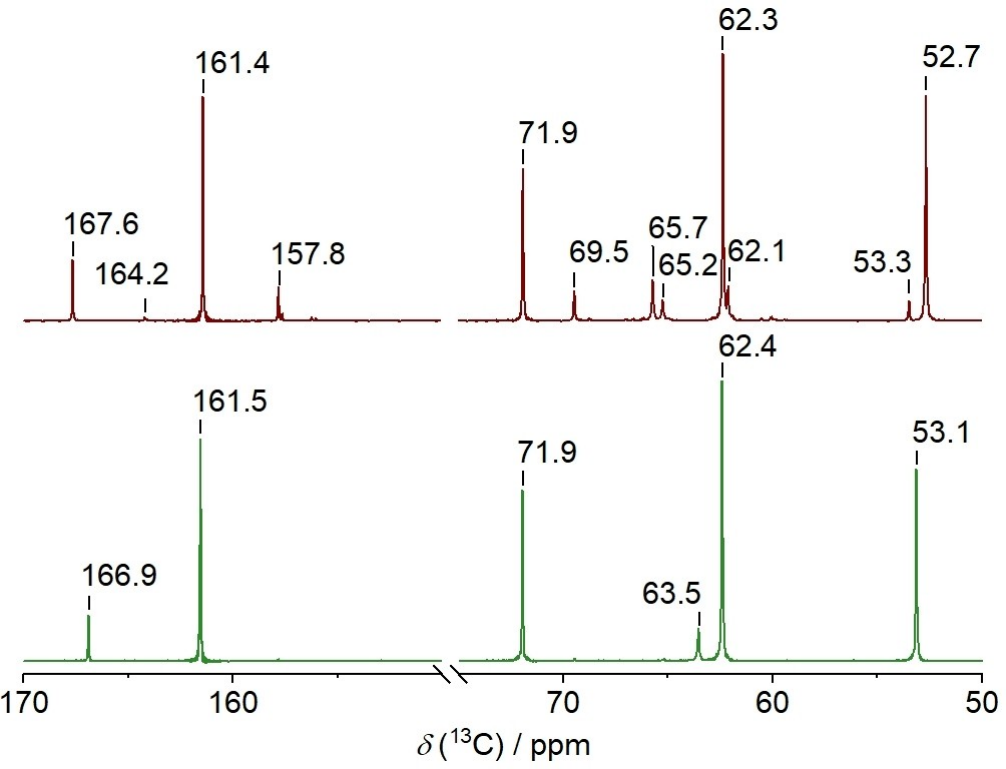
^13^C NMR spectrum of [Hbet]Cl/4U/2.5GLY before (bottom) and after (top) the storage at 60 °C for 33 days.

**Figure 2:**
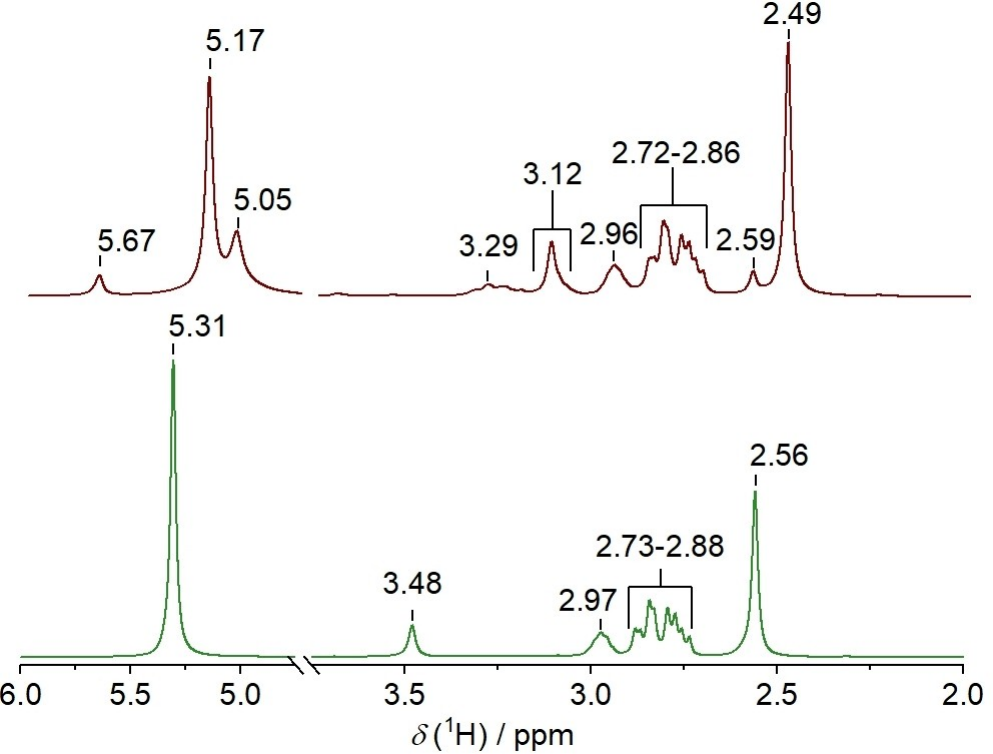
^1^H NMR Spectrum of [Hbet]Cl/4U/2.5GLY before (bottom) and after (top) the storage at 60 °C for 33 days.

The as‐prepared DES shows only the expected signals, as depicted in Figure 1 and Figure 2. No −OH signal of [Hbet]^+^ is observed in the ^1^H NMR spectrum because of typical fast proton exchange processes. The signals of the ^1^H and ^13^C NMR spectra of [Hbet]Cl/4U/2.5GLY are assigned in Table 1. In the ^1^H NMR spectrum, the intensity of the urea signal and the signal of the betainium −CH_3_ groups is expected to be 4 to 1 with the number of protons taken into account. However, the determined ratio is 6 to 1, suggesting that other groups contribute to this signal as well. This could be the −OH protons of glycerol that exchange with the ‐NH_2_ protons. Measurements at different temperatures confirm that the signal is influenced by exchange processes (Figure S1, Supporting Information).

**Table 1:** Peaks in the ^13^C (75 MHz) and ^1^H (300 MHz) NMR spectra of [Hbet]Cl/4U/2.5GLY.

Compound	Group	*δ*(^13^C) [ppm]	*δ*(^1^H) [ppm]
[Hbet]^+^	−CH_3_	53.1	2.56
Glycerol	−CH_2_‐	62.4	2.73–2.88
[Hbet]^+^	−CH_2_‐	63.5	3.48
Glycerol	−CH‐	71.9	2.97
Urea	C=O	161.4	–
[Hbet]^+^	−COOH	167.6	–
Urea	−NH_2_	–	5.31

**Scheme 1:**
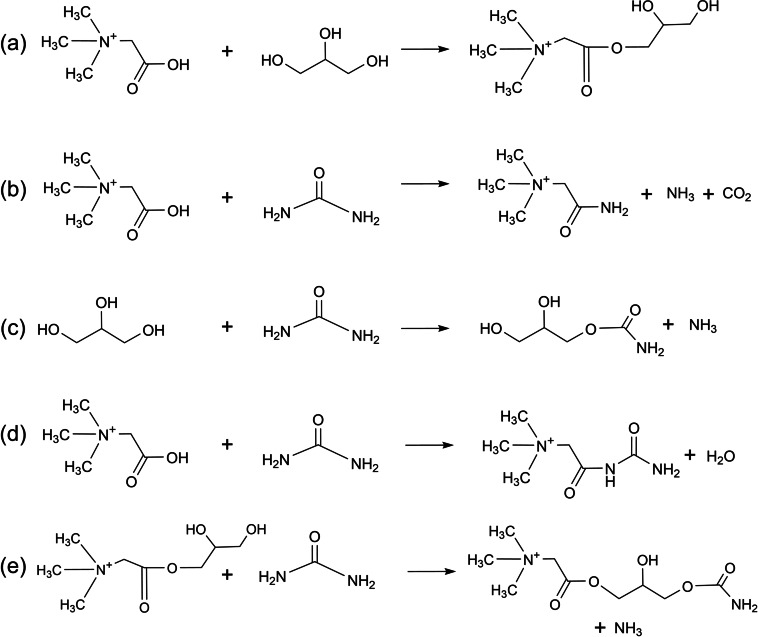
Decomposition reactions in [Hbet]Cl/4U/2.5GLY. (a) Esterification of [Hbet]^+^ and glycerol, (b) formation of a primary amide, (c) reaction of urea and glycerol, (d) condensation reaction of betaine and urea, (e) reaction of urea and betaine glycerol ester.

**Figure 3:**
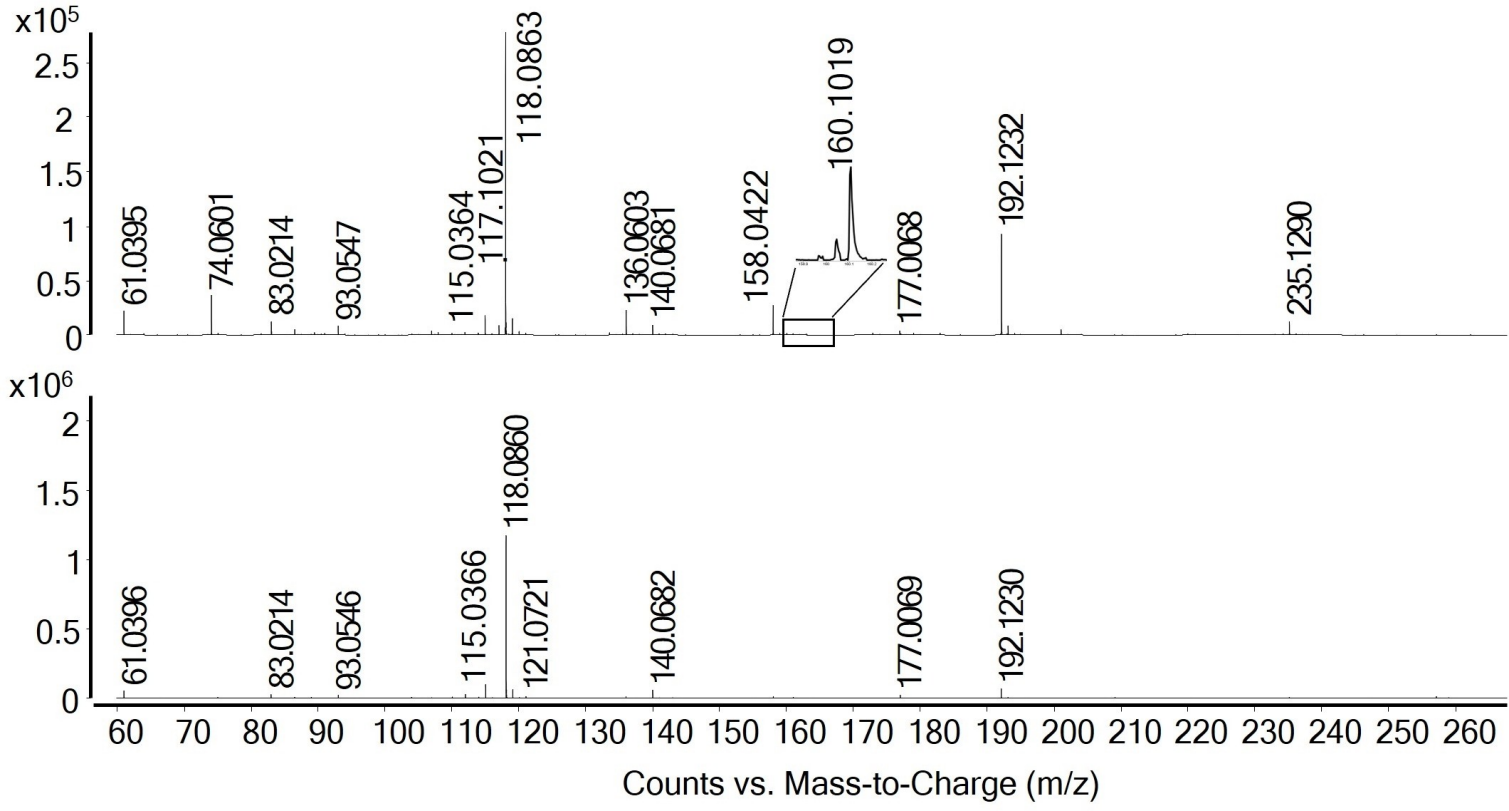
Mass spectra of as‐prepared [Hbet]Cl/4U/2.5GLY (bottom) and after storage for 33 days at 60 °C and 5 months at room temperature (top). Several low‐intensity peaks could not be assigned to molecular structures. They might result from various minor decomposition products.

The spectra significantly differ after storage at 60 °C, indicating decomposition. Conceivable reaction pathways that were previously described in literature are depicted in Scheme 1. Firstly, betaine and glycerol form an ester (Scheme 1a), as reported for other DESs containing carboxylic acids. This reaction was reported to also take place at room temperature, whereby up to 10 % of the carboxylic acid were present in esterified form directly after DES synthesis and up to 35 % after 11 months. Storage at elevated temperatures promotes this reaction.^[13]^ The signals at 53.3, 62.1, 65.2, 69.5 and 164.2 ppm in the ^13^C NMR spectrum are assigned to the product of this reaction. Corresponding ^1^H NMR signals are found at 2.59, 2.72–2.86, around 3.12, and 3.29 ppm. The product was verified by mass spectrometry (*m/z* [M]^+^ 162.1232, Figure 3 and Table 2) and also found in the mass spectrum of the as‐prepared DES. This shows that esterification already starts during DES preparation. Another signal at *m/z* [M]^+^ 235.1290 is assigned to the reaction product of this betaine glycerol ester with urea (Scheme 1e). As especially ^13^C NMR measurements have a lower sensitivity, not all signals of the reaction products that were detected by mass spectrometry can be observed.

**Table 2:** Assignment of the mass spectra peaks in Figure S1.

Substance	Detected ion	m/z	As‐prepared DES	DES stored at 60 °C
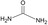	[M+H]^+^	61.0396	yes	yes
	[M+Na]^+^	83.0214	yes	yes
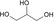	[M+H]^+^	93.0547	yes	yes
	[M+Na]^+^	115.0366	yes	yes
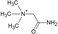	[M]^+^	117.1021	no	yes
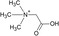	[M]^+^	118.0863	yes	yes
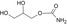	[M+H]^+^	136.0603	no	yes
	[M+Na]^+^	158.0422	no	yes
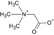	[M+Na]^+^	140.0682	yes	yes
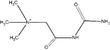	[M]^+^	160.1079	no	yes
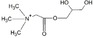	[M]^+^	192.1232	yes	yes
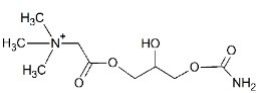	[M]^+^	235.1290	no	yes

Furthermore, betaine and urea appear to form a primary amide according to Scheme 1b, as indicated by the additional signal at 5.67 ppm in the ^1^H NMR spectrum in agreement with mass spectrometry (*m/z* [M]^+^ 117.1021) and literature.^[46]^ Thereby, CO_2_ and NH_3_ evolve, explaining the ammonia odor also noticed when the DES was heated only below the decomposition temperature of urea (133 °C).^[31]^

The reaction of urea and glycerol (Scheme 1c) is indicated by the signal at 5.05 ppm in the ^1^H NMR and at 157.8 ppm in the ^13^C NMR spectra as well as the signal at *m/z* [M+H]^+^ 136.0603 in the mass spectrum. This glycerol urethane is an intermediate of the formation of glycerol carbonates.^[47]^ However, no signals in NMR or mass spectra were found that would suggest the formation of the cyclized product.

In the mass spectrum, a weak signal at *m/z* [M]^+^ 160.1079 indicates the condensation reaction of betaine and urea (Scheme 1d). However, the reaction was not verified by NMR spectroscopy due to the absence of an NH signal expected in the range of 8–10 ppm.^[48]^ A very low concentration of the product may hinder the detection by NMR spectroscopy.^[48]^

Altogether, the ^1^H and ^13^C NMR and mass spectra indicate several parallel decomposition reactions taking place even at 60 °C. All three components of the DES take part in these processes. Thereby, the ^13^C NMR signals of reacted betainium, glycerol and urea could be assigned to more than one of the discussed decomposition products. Furthermore, the overlapping signals of reagents and products in the ^1^H NMR spectrum impede quantification. However, the absence of NMR signals of reaction products **1 d** and **1 e** allows the conclusion that decomposition processes **1 a**–**c** dominate.

Therefore, in the following, the term [Hbet]Cl/4U/2.5GLY is used knowing that it is inaccurate as the decomposition reactions affect the molar ratios of [Hbet]Cl, urea and glycerol and add additional compounds to the DES mixture.

Another compound that has to be taken into account when working with hydrophilic DESs, such as [Hbet]Cl/4U/2.5GLY, is water. The water content of a freshly prepared DES was determined by Karl Fischer titration to be 0.14–0.25 wt.%, highlighting the hygroscopy of the DES. Such small amounts of water are not necessarily expected to be detected by ^1^H NMR spectroscopy due to low signal intensity and signal broadening caused by intermolecular interactions. In a previous study on the DES ChCl/2U, it was found that water at less than 1 wt.% contributes to the hydrogen bond network and only at higher levels do disruptive effects occur.^[49]^ Although the amount of water contained in freshly prepared [Hbet]Cl/4U/2.5GLY might not interfere with the DES nanostructure, it should be considered as another minor component potentially influencing the DES properties. In addition, an accumulation of water must be expected if the DES is stored in air or if water is formed during a chemical reaction.

### Metal oxide dissolution in [Hbet]Cl/4U/2.5GLY

In order to investigate the metal oxide dissolution ability of [Hbet]Cl/4U/2.5GLY qualitatively, 30 mostly water‐insoluble metal oxides were heated to 150 °C in the DES at a molar ratio of *n*_M_ : *n*_DES_=1 : 4 in a flask open to the air (*n*_DES_≡*n*_[Hbet]_). The reaction conditions were chosen to allow a relatively close comparison to previous experiments in the IL [Hbet][NTf_2_]. In [Hbet]Cl/4U/2.5GLY, [Hbet]^+^ is expected to be the strongest ligand for metal ions due to the chelating abilities of the carboxylic group. Therefore, the comparison to the IL [Hbet][NTf_2_] is obvious. Since previous solubility studies in the IL^[40]^ involved chloride‐induced betainium decomposition at 175 °C,^[4]^ that can be avoided by a lower reaction temperature of 150 °C,^[10]^ this temperature was chosen for experiments in [Hbet]Cl/4U/2.5GLY. At 150 °C, water evolving during the reaction is expected to partly evaporate, but due to the hydrophilic nature of the DES, a certain water content should always be considered.

However, chloride could also play a significant role, as previous investigations on cobalt oxide dissolution showed.^[10]^ Since [Hbet]Cl/4U/2.5GLY is a chloride‐rich solvent, metal oxide solubilities were compared to an equimolar mixture of [Hbet][NTf_2_] and [Hbet]Cl, that is, [Hbet]_2_[NTf_2_]Cl,^[40]^ instead of the binary IL. It has to be taken into account that, although the investigations in IL were also performed with molar ratios of *n*_M_ : *n*_[Hbet]_=1 : 4, the metal‐chloride ratio in the IL is only *n*_M_ : *n*_Cl_=1 : 2, that is, half as large as in the DES, which might affect solubility.

Table 3 gives an overview about the solubility of 30 metal oxides in [Hbet]Cl/4U/2.5GLY and in [Hbet]_2_[NTf_2_]Cl. While metal oxides dissolved in [Hbet]_2_[NTf_2_]Cl could be detected by IR spectroscopy due to a shift of the asymmetric stretching vibration of the [Hbet]^+^ carboxyl group, this is not possible for DES samples. Overlapping bands of the DES components in the range 1500–1800 cm^−1^ reduce the significance of the IR spectra.^[45]^ Therefore, the following conclusions are based on visual observations and PXRD studies of the residual powders after removal of the DES by dissolution in water and subsequent centrifugation. In order to get a rough quantitative estimate, the remaining solids were weighted after drying. This is only a guideline as the starting materials were often very fine powders that did not settle completely during centrifugation and losses must be considered. The mass fractions of the recovered solids as well as more information on experimental observations and PXRD are given in Table S1 and Figures S2 and S3.

**Table 3:** Overview about the qualitative solubility of 30 metal oxides in the DES [Hbet]Cl/4U/2.5GLY at 150 °C compared to the IL [Hbet]_2_[NTf_2_]Cl at 175 °C.^[40]^

Metal oxide	Solubility in DES	Comment	Solubility in IL
Al_2_O_3_	Negligible	–	Negligible
BaO	Full	Clear, colorless solution after 5 min	Full, subseq. BaCl_2_ precipitation
Bi_2_O_3_	Negligible	(BiO)_2_CO_3_ formation instead of dissolution	Full, subseq. BiOCl precipitation
CaO	Full	Clear, colorless solution after 10 min	Full
CoO	High	Rapid blue coloration of solution, still undissolved reagent after 24 h	Full
Co_3_O_4_	High	Rapid blue coloration of solution, still undissolved reagent after 24 h	Full
Cr_2_O_3_	Negligible	–	Negligible
Cu_2_O	Full	Dark blue solution after 45 min	Full, subseq. CuCl precipitation
CuO	Full	Dark blue solution after 2 h, blue precipitate	Full
Fe_2_O_3_	Negligible	–	Low
FeOOH	Negligible	–	Full
Ga_2_O_3_	Negligible	–	Negligible
GeO_2_	Full	Clear, colorless solution after 1 h	Negligible
LiCoO_2_	High	Rapid blue coloration of solution, still undissolved reagent after 24 h	Full
MgO	Full	Clear, colorless solution after 1 h	Full
MnO	Full	Clear, slightly brownish solution after 10 min	Full
MnO_2_	High/Full	Depending on MnO_2_ purity brown solution after 3 h	Full
MoO_3_	Full	Brown solution after 1 h	Low
NiO	Negligible	–	Low
PbO	Full	Clear, colorless solution after 10 min	Full, subseq. PbCl_2_ precipitation
PbO_2_	Full	Clear, yellow solution after 10 min	Full
Sb_2_O_3_	Negligible	–	High
SnO	High	Dissolution and subsequent Sn_6_O_4_(OH)_4_ precipitation ageing to SnO	Full
SrO	Full	Clear, colorless solution after 10 min	Full
TiO_2_	Negligible	–	Negligible
V_2_O_3_	Low	–	High
VO_2_	Low	–	–
V_2_O_5_	Full	Dissolution and subsequent precipitation of amorphous solid	Full
WO_3_	Low	–	Negligible
ZnO	Full	Clear, colorless solution after 5 min	Full

**Al_2_O_3_**, **Cr_2_O_3_**, **Ga_2_O_3_**, and **TiO_2_** are essentially insoluble in the DES and in the IL. In contrast to the IL, **Fe_2_O_3_**, **FeOOH**, **NiO** and **Sb_2_O_3_** are also not soluble in the DES, which is ascribed to the differing pH. [Hbet]^+^ is a moderately strong acid with a p*K*_a_ value of 1.86.^[50]^ In the DES, the acidic proton, on the one hand, is involved in the hydrogen bond network, on the other hand, evolving ammonia increases the pH. This was confirmed by placing moist pieces of universal pH indicator paper above multiple reaction flasks. A rapid dark blue coloration indicated the evaporation of basic vapors. This is in agreement with Fe_2_O_3_ and FeOOH being soluble in acids, but only poorly soluble in neutral or basic media.^[51]^ Similarly, the acidity of the DES might be too low for the dissolution of basic NiO^[1]^ and the amphoteric Sb_2_O_3_.^[1]^ The negligible solubility of the latter three metal oxides was verified by testing the aqueous liquid phase of each sample with typical detecting reactions, namely the addition of KSCN or K_4_[Fe(CN)_6_] for iron(III), dimethylglyoxime for nickel(II) and Na_2_S for antimony(III). None of the typical color changes or precipitates expected in the presence of the respective ions (see Experimental Section) was observed.

Complete dissolution of **BaO**, **CaO**, **Cu_2_O**, **CuO**, **MgO**, **MnO**, **MnO_2_**, **PbO**, **PbO_2_**, **SrO** and **ZnO** is observed in the DES as well as in the IL. Among them, BaO, CaO, MnO, PbO, PbO_2_, SrO and ZnO dissolve very fast in the DES (within minutes as listed in Table 3). A reason could be a higher water content in the DES, which clearly facilitates the dissolution reaction.[^10^, ^36^] MnO_2_ behaves differently with purity: 85–90 % pure MnO_2_ dissolves completely within 3 h, forming a brown solution. In contrast, 98 % pure MnO_2_ dissolves at a much slower rate, and traces of black powder (MnO_2_ and something unidentified) remained after a reaction time of 24 h. In order to rule out a mere particle size effect, both MnO_2_ reagents were ground the same way before usage. A similar impurity effect has been reported for dissolving CuO in [Hbet][NTf_2_].^[4]^

After the dissolution of Cu_2_O, BaO and PbO in the IL, the respective chlorides precipitate, which does not happen in the DES. It is known that CuCl forms soluble complexes with amine ligands, in which copper(I) is subsequently oxidized by atmospheric oxygen to copper(II).^[1]^ Urea or evolving ammonia appear to act in this manner, as copper(II) is indicated by a blue coloration of the DES solution. This was confirmed by the reaction Cu_2_O in [Hbet]_2_[NTf_2_]Cl together with urea or ammonia. Both samples yielded dark blue solutions within 10 min, in contrast to a sample without urea or ammonia. This indicates the beneficial effect of amine ligands. When Cu_2_O is dissolved in [Hbet]Cl/4U/2.5GLY under inert atmosphere, the solution initially colors slightly blue and is light yellow after 4 h. This is ascribed to traces of oxygen or other oxidizing agents that oxidize small amounts of copper(I) to copper(II). When the sample is exposed to air, the color intensifies rapidly to dark blue, highlighting the oxidizing effect of atmospheric oxygen.

As BaCl_2_ is well soluble in water, it might be dissolved in the hydrophilic [Hbet]Cl/4U/2.5GLY, but precipitate from the hydrophobic [Hbet]_2_[NTf_2_]Cl. Indeed, the water content measured for [Hbet]Cl/4U/2.5GLY directly after synthesis (0.14‐0.25 wt.%) is significantly larger than reported for [Hbet][NTf_2_] (35 ppm).^[36]^ On the other hand, the poorly water‐soluble PbCl_2_ is known to form soluble [PbCl_3_]^−^ and [PbCl_4_]^2−^ complexes in the presence of excess chloride,^[1]^ as present in the DES but not in the IL. Furthermore, both barium and lead are known to form glycerolate complexes, that might prevent precipitation.[^52^, ^53^, ^54^]

When **Bi_2_O_3_** is heated in the DES, it quickly forms a voluminous white powder, which after 18 h yields a mass that can no longer be stirred. Considering the broad PXRD reflections (Figure S2), the solid is identified as a mixture of the starting material and (BiO)_2_CO_3_. It has been shown that Bi_2_O_3_ nanoparticles dispersed in water can capture CO_2_ from the air and form bismuth subcarbonates. Thereby, the acidic CO_2_ is absorbed on the basic Bi_2_O_3_ particle surface in a heterogeneous reaction. CO_2_ is also evolved by the amide formation of betaine and urea (Scheme 1b). In this process, bismuth(III) is not assumed to go into solution since no BiOCl formation is observed, which is expected for bismuth(III) ions dissolved in the presence of chloride.^[55]^ This is in contrast to the reaction in [Hbet]_2_[NTf_2_]Cl, where BiOCl precipitates. Due to the lower acidity of [Hbet]Cl/4U/2.5GLY, the reaction with acidic CO_2_, potentially catalyzed by the DES, seems to be preferred over the dissolution of Bi_2_O_3_.

The cobalt oxides **CoO**, **Co_3_O_4_** and **LiCoO_2_** show similar reactivity in the DES and the IL deducing from the fast color change of the liquid phase to blue. The incomplete dissolution in the DES could be ascribed to the lower reaction temperature. The dissolution of LiCoO_2_ in chloride‐containing [Hbet][NTf_2_] was also shown to be incomplete after 24 h stirring at 150 °C in an open flask.^[10]^ The used CoO and Co_3_O_4_ contained a small amount of Co_3_O_4_ and CoO, respectively, whose reflections are also observed in the diffractogram of the residual powder.

Unlike in the IL, the used amount of **SnO** did not completely dissolve in the DES. Instead, a white solid precipitated within 1 h, which increasingly darkened with longer reaction times. SnO is assumed to dissolve in [Hbet]Cl/4U/2.5GLY and to subsequently form Sn_6_O_4_(OH)_4_ which is known to age to SnO.^[56]^ This is in agreement with the sample diffractograms after 1 h and after 24 h (Figure S5). Thus, it is essentially a recrystallization of SnO. Although the mass of the precipitate is almost the same as of the starting material, a considerable amount of solvated tin(II) is assumed to be present in the liquid phase, as shown in the following section. A contribution to the powder mass is expected from a compound generating unidentified reflections in the PXRD and potentially from an amorphous solid.

The vanadium oxides **V_2_O_3_**, **VO_2_** and **V_2_O_5_** also dissolve in the DES as the greenish blue color of the solutions in the first minutes of reaction indicates. In an aqueous solution, the color would hint at a mixture of the aqua‐complexes of vanadium(III) and vanadium(IV). Vanadium is known to readily change its oxidation state and mixtures of V_2_O_3_ and the oxidized compound V_3_O_5_, or VO_2_ and the reduced oxide V_6_O_13_, were identified in the starting materials as well as the solid residuals by PXRD (Figure S4). After 24 h of reaction, a brown color of the solution indicates decomposition of the DES. The dark brown powder obtained from the dissolution of V_2_O_5_ in the DES is an amorphous vanadium compound that precipitated following the dissolution of V_2_O_5_.

Unlike [Hbet]_2_[NTf_2_]Cl, the DES dissolves **GeO_2_** and **MoO_3_** completely and **WO_3_** partially. The urea component or the evolving ammonia seem to be crucial, because GeO_2_, MoO_3_ and WO_3_ are only poorly soluble in acids, but dissolve well in basic media.^[1]^ This was confirmed by a control experiment in which dissolution of GeO_2_ in a solution of [Hbet]Cl and glycerol was attempted at 150 °C. No reaction was observed within 3 h, while the addition of urea or ammonia led to complete dissolution within 20 min or a few seconds, respectively.

Since the WO_3_ sample still contained unreacted starting material after 24 h, the presence of dissolved tungsten(VI) was verified by a typical analytical reaction, whereby a blue color, characteristic for tungsten blue, was observed upon the addition of diluted sulfuric acid and tin powder to the aqueous liquid phase (see Experimental Section and Figure S6). The higher solubility of MoO_3_ compared to WO_3_ can be related to the crystal structures: WO_3_ is a three‐dimensionally cross‐linked framework, MoO_3_ is a layered structure.^[1]^ This is consistent with the solubility of the two oxides in water, which is low for MoO_3_ but negligible for WO_3_.^[57]^

In summary, the dissolution performance of [Hbet]Cl/4U/2.5GLY is similar to [Hbet]_2_[NTf_2_]Cl for many metal oxides. Much of the variation can be attributed to the increase in pH due to ammonia produced by the decomposition of the urea component of the DES.

### Solubility limits for the example of zinc

After the above‐described experiments gave insights into the qualitative solubility of various metal oxides in [Hbet]Cl/4U/2.5GLY, we were interested in the maximum quantity that can be dissolved. Since this is well investigated for ZnO in [Hbet][NTf_2_],[^9^, ^58^] the same metal oxide was chosen here.

As expected, a temperature‐dependent dissolution behavior was observed, yet not a trivial one. At 150 °C, complete dissolution is observed at molar ratios up to *n*_ZnO_ : *n*_DES_=1 : 1 within 1 h. At 120 °C, the same mixture forms a white, foam‐like solid on top of a gel‐like, hardly stirrable, turbid mixture. The white solid is a mixture of ZnO and zinc glycerolate, that is, a precipitate with a component of the DES (Figure S7). At half the concentration (*n*_ZnO_ : *n*_DES_=1 : 2), ZnO dissolves at 120 °C within 10 min. Mixtures with the intermediate concentration *n*_ZnO_ : *n*_DES_=1 : 1.5 form a clear, yellowish liquid within 1 h, but the liquids remain turbid with higher zinc contents even after 24 h.

Mechanistically, the preferred formation of [Zn(bet)_2_]^2+^ complexes is assumed, in analogy with previous investigations in [Hbet][NTf_2_].^[58]^ The DES can provide these favorable interactions to 0.5 eq. zinc(II) regardless of the reaction temperature. At 120 °C, additional amounts of ZnO exceeding *n*_ZnO_ : *n*_DES_=1 : 2 could be dissolved more slowly by interactions with ammonia formed even below the decomposition temperature of urea (Scheme 1b, c, e) or with glycerol, taking heteroleptic complexes into account. The formation of zinc glycerolate in samples with *n*_ZnO_ : *n*_DES_=1 : 1 matches the previously observed formation of this compound when ZnO is heated in glycerol above 110 °C.[^52^, ^59^, ^60^] On the other hand, at 150 °C, urea quickly decomposes, forming ammonia. Thus, additional zinc(II), exceeding *n*_ZnO_ : *n*_DES_=1 : 2, could be coordinated by forming [Zn(NH_3_)_4_]^2+^.^[1]^ As one ammonia molecule evolves per two equivalents of urea upon decomposition, [Hbet]Cl/4U/2.5GLY provides 2 eq. NH_3_ besides 1 eq. [Hbet]^+^. This would equal the coordination of 0.5 eq. Zn^2+^ in [Zn(bet)_2_]^2+^ and another 0.5 eq. Zn^2+^ bound in [Zn(NH_3_)_4_]^2+^. This matches the observation that the dissolution of 0.5 eq. ZnO takes place within 10 min, while the exceeding amounts require significantly longer times (1 h). We assume that, thereby, ammonia ligands are continually provided by the proceeding decomposition of urea.

When it comes to large‐scale metal production, the amount of necessary auxiliary materials, such as solvents or electrolytes, significantly affects the economic feasibility of the whole process. Even when such auxiliaries can be recycled, large quantities result in larger reaction facilities, heating expenses and storage volumes. In order to reduce solvent amounts to the minimum, high metal concentrations are required. Conventional zinc electrolysis is performed with aqueous solutions with 46–90 g L^−1^ zinc, resulting in a concentration of 0.7–1.4 mol L^−1^.[^61^, ^62^] The highest conductivity of a ZnSO_4_ electrolyte for supercapacitors was found at a zinc concentration of 2 mol L^−1^.^[63]^ Solutions of ZnO dissolved in [Hbet]Cl/4U/2.5GLY or [Hbet][NTf_2_] can almost keep up with these concentrations. Thus, ZnO dissolves in [Hbet][NTf_2_] up to a molar ratio of 1 : 2, amounting to a concentration of 1.9 mol L^−1^ using the density of the IL at 60 °C (1.531 g cm^−3^) as reported by Nockemann et al.^[36]^ As the dissolution of ZnO in [Hbet]Cl/4U/2.5GLY exceeding *n*_ZnO_ : *n*_DES_=1 : 2 involves longer reaction times or higher temperatures and, therefore, significant decomposition, we consider this molar ratio most suitable for this discussion. Thus, the amount of soluble ZnO equals a concentration of 1.0 mol L^−1^ with the density of the DES at 60 °C (1.2411 g cm^−3^) reported by Kuperkar et al. as basis.^[45]^ Hence, both [Hbet]Cl/4U/2.5GLY and [Hbet][NTf_2_] could compete with aqueous ZnSO_4_ electrolytes, but higher concentrations are achievable in [Hbet][NTf_2_], which could be beneficial for efficient future processes.

### Electrodeposition in [Hbet]Cl/4U/2.5GLY

**Figure 4:**
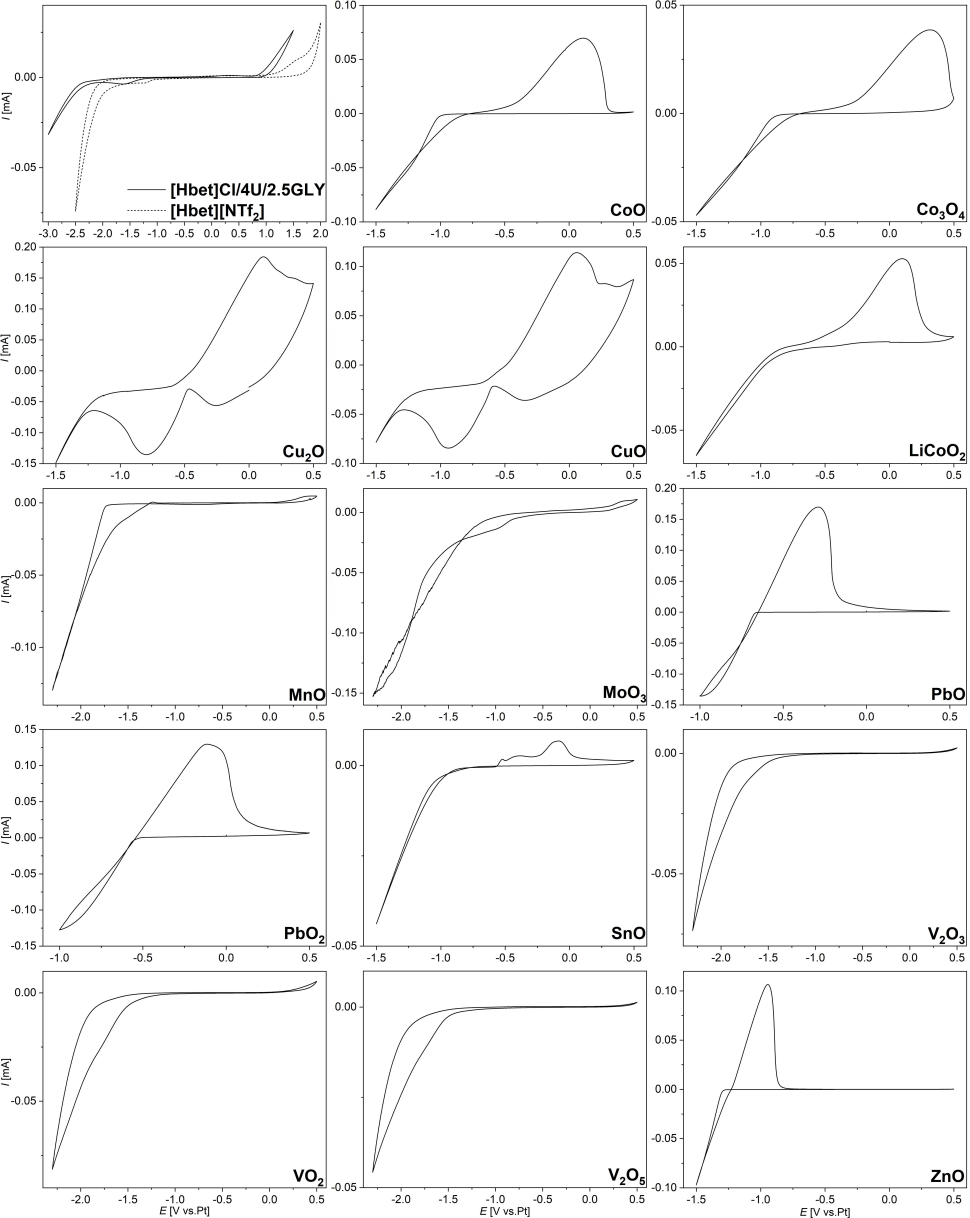
CVs of [Hbet]Cl/4U/2.5GLY compared to [Hbet][NTf_2_] as well as CoO, Co_3_O_4_, Cu_2_O, CuO, LiCoO_2_, MnO, MoO_3_, PbO, PbO_2_, SnO, V_2_O_3_, VO_2_, V_2_O_5_ and ZnO dissolved in [Hbet]Cl/4U/2.5GLY (*n*_M_ : *n*_DES_=1 : 8) recorded at 60 °C and a scan rate of 10 mV s^−1^.

An important part of ionometallurgy is the recovery of dissolved metals. We here investigate the applicability of an electrochemical approach. CVs were recorded in a three electrode electrochemical cell with glassy carbon, a platinum plate and a platinum wire as working, counter and pseudo‐reference electrode, respectively. The samples were kept at 60 °C in order to avoid crystallization during the measurement. CV of pure [Hbet]Cl/4U/2.5GLY suggests that the DES is stable in the range −2.3 V to 0.9 V measured against a platinum wire pseudo‐reference electrode, as shown in Figure 4. This slightly differs from the electrochemical window of [Hbet][NTf_2_], −2.0 V to 1.4 V, measured in the same set‐up.^[9]^ The measurements were performed without ferrocene as internal reference due to previously reported interactions with traces of water in the IL [Hbet][NTf_2_].^[10]^ As [Hbet]Cl/4U/2.5GLY is hygroscopic and cannot be dried at elevated temperatures without promoting decomposition, the water content is even higher than in the referenced IL. Therefore, the limited comparability of numerical values should be noted. Both [Hbet]Cl/4U/2.5GLY and [Hbet][NTf_2_] show very small oxidation and reduction signals within their electrochemical stability range that might originate from impurities such as water.

Electrochemical investigations were performed for samples in which the metal oxide either completely dissolved or intense coloring indicated considerable dissolution despite some unreacted starting material. Due to the very negative reduction potentials of the alkaline earth metals, these were not investigated. CoO, Co_3_O_4_ Cu_2_O, CuO, LiCoO_2_, MnO, MoO_3_, PbO, PbO_2_, SnO, V_2_O_3_, VO_2_, V_2_O_5_ and ZnO were stirred in [Hbet]Cl/4U/2.5GLY (*n*_M_ : *n*_DES_=1 : 8) at 150 °C until no solid was observed anymore or, in the case of the cobalt and vanadium oxides as well as SnO, for 4 h. At longer reaction times, a strong color change to yellow and brown was observed, indicating severe decomposition of the DES. After CV measurements, the metals were attempted to be electrodeposited on a carbon cloth working electrode by applying a certain potential for 1 h. From our experience, carbon cloth has the advantage that potentials of specific reactions are similar to glassy carbon. Therefore, the previously measured CV could be used as rough orientation to choose a suitable deposition potential.

The CVs of all samples, except for MnO, MoO_3_ and the vanadium oxides, show reduction and oxidation signals within the electrochemical window of the DES, as presented in Figure 4. They are attributed to the reduction of the respective metal ion to the element and subsequent metal stripping. In agreement with that, metals were deposited on carbon cloth and identified by EDX (Figures S8 to S16).

Typical for copper,^[64]^ two reduction and oxidation peaks, respectively, indicate the stepwise reaction from copper(II) over copper(I) to copper(0) and vice versa. Another small oxidation peak around 0.4 V might result from a reaction of DES components, for example, water, catalyzed by copper ions. The very similar CVs for solutions of Cu_2_O and CuO as well as for solutions of PbO and PbO_2_ indicate that reduction starts from the same oxidation state, namely copper(II) or lead(II). While the presence of copper(II) is obvious from the blue color of both samples, the presence of Pb(II) was confirmed by the addition of aqueous Na_2_CO_3_ solution, upon which PbCO_3_ precipitated from both solutions. Thus, the dissolution could involve a reaction with atmospheric oxygen or the DES acts as a redox‐active solvent and is partially converted during the dissolution process. The same redox reactions were observed upon the dissolution of Cu_2_O and PbO_2_ in [Hbet][NTf_2_].[^40^, ^41^]

In contrast to all other CVs, the solutions of MnO, MoO_3_ and vanadium oxides only show a reduction signal, but no oxidation peak. This could indicate irreversible metal deposition or another reduction reaction, that is, the decomposition of the DES, potentially catalyzed by the metal ions. As manganese and molybdenum are not deposited on carbon cloth, but gas evolves during potential application, the second scenario is more likely. Small isolated particles of vanadium were electrodeposited from V_2_O_3_, VO_2_ and V_2_O_5_ solutions (Figures S17 to S19), however, this was only possible at a potential close to the edge of the electrochemical window of [Hbet]Cl/4U/2.5GLY. Thus, DES decomposition must be taken into account, which is supported by the observation of gas evolution.

In summary, cobalt, copper, zinc, tin, and lead can be electrodeposited from [Hbet]Cl/4U/2.5GLY as well as very small amounts of vanadium. Although the CV of the pure DES suggests a cathodic stability range up to −2.3 V, electrochemical decomposition reactions appear to take place at less negative potentials with metal ions dissolved. Therefore, the electrochemical long‐term stability should be investigated individually for each sample. In the chosen set‐up, numerous side products have to be expected as various reactions can take place at the anode. These side reactions might be avoidable or reducible by the use of sacrificial agents or separated cell compartments,^[34]^ however, this does not appear worthwhile with the DES already forming decomposition products during its synthesis and metal oxide dissolution.

## Conclusions

The test on the applicability of the DES [Hbet]Cl/4U/2.5GLY for ionometallurgical metal extraction demonstrates very good solubility for several metal oxides and also allows for metal electrodeposition of cobalt, copper, lead, tin, zinc and, to a small extent, vanadium. However, its applicability to industrial processes is highly questionable due to its thermal and chemical instability. Although some decomposition products were shown to support the dissolution of metal oxides, such as Cl_3_^−^ in the case of LiCoO_2_,^[65]^ other side reactions (see Scheme 1) and its redox‐activity towards some of the metal oxides reduce the performance of the DES. Thus, it is unsuitable for many subsequent cycles of metal oxide dissolution and metal deposition. In addition, chemical recycling will be challenging with numerous (partly unidentified) volatile and in part also toxic by‐products. Considering this aspect, [Hbet]Cl/4U/2.5GLY cannot be called a green solvent and these findings reveal that the thorough understanding of the fundamental chemistry is crucial before any solvents can be applied in seemingly sustainable processes.

Comparing the solubility in [Hbet]Cl/4U/2.5GLY and [Hbet]_2_[NTf_2_]Cl again highlights acidity or basicity as crucial factor for metal oxide dissolution, as previously discussed for [Hbet][NTf_2_].^[40]^ For example, the significantly better solubility of GeO_2_, MoO_3_ or WO_3_ in [Hbet]Cl/4U/2.5GLY, that is ascribed to ammonia evolution, identifies the strong acid [Hbet]^+^ as an unsuitable reaction partner for acidic or amphoteric metal oxides. Instead, a stable basic IL or DES should be chosen. It is also conceivable to introduce an in‐situ ammonia source into an IL or DES with convenient properties, for example, a large electrochemical window. This principle can be systematically exploited to selectively dissolve components from mixtures such as primary or secondary resources. Future research should consider the acid‐base and the redox properties of each individual metal oxide and choose a solvent accordingly.

Furthermore, when DESs are considered for ionometallurgy, more attention should be paid to the effects of dissolved components on the hydrogen bond network. Although DES components are usually clearly labeled as HBD and HBA, the situation is more complex when it comes to carboxylic acids, which can act both as HBD and HBA. The dissolution of metal oxides, removing carboxylic hydrogen from the DES system and forming metal‐carboxylate complexes (and also polyol complexes), therefore, might significantly affect hydrogen bonding. Since the intermolecular interactions are crucial for the character of a DES, a high concentration of metal cations is expected to strongly affect the fundamental DES properties. Calculations, simulations and vapor pressure measurements could resolve this in more detail.

The decomposition reactions taking place in [Hbet]Cl/4U/2.5GLY at room temperature and, much more strongly, at increased temperatures will have a similar effect on the hydrogen bond network. Therefore, chemical and thermal stability of the DES and the DES‐based solution must be achieved.

At the current state of ionometallurgical research, neither ILs nor DESs can be considered perfect and general solvent systems. ILs are usually more stable, but often rely on problematic and costly chemicals. Many DESs, on the other hand, are easily produced from renewable resources, but degradation reactions prevent their use. Moreover, due to their decomposition, they cannot be unreflectively classified as green solvents, solely on the basis of their components. Thus, the application in ionometallurgical processes could aim at a favorable combination of ILs and DESs in order to exploit their advantages and minimize their disadvantages.

Specifically [Hbet]Cl/4U/2.5GLY does not appear to be a suitable DES for further ionometallurgical application. However, the insights gained during its investigation can contribute to the development of stable and green solvents. We believe this is a task worth solving, even if the disruptive innovation ultimately required in the metal‐producing industry will be a major challenge.^[66]^

## Experimental Section

**Chemicals**. [Hbet]Cl (99 %), urea (99.3 %), CoO (99.995 %), FeOOH (99.8 %), Ga_2_O_3_ (99.99 %), GeO_2_ (99.999 %), LiCoO_2_ (99.5 %, <0.1 % Ni), MoO_3_ (99.999 %), PbO (99.999 %), Sb_2_O_3_ (99.6 %), SrO (99.50 %), V_2_O_3_ (95 %) and VO_2_ (99 %) were purchased from abcr. BaO (97 %), Fe_2_O_3_ (p. a.) and V_2_O_5_ (p. a.) were obtained from Riedel de Haën, Bi_2_O_3_ (99 %), Cr_2_O_3_ (98 %), Cu_2_O (99 %), CuO (99.995 %) and PbO_2_ (97 %) from Alfa Aesar. SnO (p. a.), TiO_2_ (99 %) and MnO_2_ (85–90 %) were obtained from Merck. MnO_2_ of higher purity (98 %) was purchased from Thermo Fisher. Both MnO_2_ reagents were ground in an agate mortar for two minutes before usage. Glycerol (99 %) was obtained from Honeywell, Al_2_O_3_ (99.7 %) from Acros Organics, CaO (p. a.) from Reachim, Co_3_O_4_ (p. a.) from Chempur, MgO (97 %) and MnO (99 %) from Aldrich, NiO (p. a.) from VEB Berlin‐Chemie, ZnO (Pharma 4) from Grillo and ammonia (25 %, p.a.) from VWR. All chemicals were stored and all reactions performed under ambient conditions, except for SrO that was kept in an argon‐filled glove box (MBraun; *p*(O_2_)/*p* ^0^<1 ppm, *p*(H_2_O)/*p* ^0^<1 ppm). Note that CoO, Co_3_O_4_, V_2_O_3_ and VO_2_ contain small amounts of Co_3_O_4_, CoO, V_3_O_5_ and V_6_O_13_, respectively, as shown in Figure S4.

**DES synthesis**. [Hbet]Cl/4U/2.5GLY was synthesized by stirring [Hbet]Cl, urea and glycerol in a molar ratio of 1 : 4 : 2.5 at 80 °C in a closed Schott bottle until a clear solution was obtained, typically two to five hours. Following this, Karl Fischer titration was performed on a TitroLine *KF trace* titrator. For subsequent analysis or experiments, the DES was synthesized on the previous day at the earliest. For NMR spectroscopy, a batch of DES was stored at 60 °C for 33 days in a closed Schott bottle under air atmosphere.

**Metal oxide dissolution**. A metal oxide and [Hbet]Cl/4U/2.5GLY were stirred in a molar ratio of *n_M_* : *n*_[Hbet]Cl_=1 : 4 at 150 °C in a flask open to the air. Samples were based on 1 g DES. The reaction was terminated when no solid powder was observable or after 24 h. For analysis, the liquid phase was dissolved in water and the solid residues centrifuged, washed with water two more times and with ethanol one time before drying in a vacuum drying cupboard at room temperature. ZnO was also dissolved in [Hbet]Cl/4U/2.5GLY with molar ratios ranging from *n*_ZnO_ : *n*_[Hbet]Cl_=1 : 2 to 1 : 1 at 120 °C or 150 °C. PXRD samples were prepared by applying the suspension on the sample holder without previous washing of the solid.

**Detection reactions**. After the reaction as described above, the FeOOH, NiO, Sb_2_O_3_ and WO_3_ samples were diluted with 3 mL water each and separated from undissolved solid by centrifugation.

(I) For iron(III) testing, the respective aqueous solution was acidified with diluted hydrochloric acid before adding three drops of KSCN or K_4_[Fe(CN)_6_] solution. In the presence of iron(III) ions, an intense red color would be observed in the first case and a dark blue precipitate in the second.

(II) For nickel(II) testing, the pH of the solution was increased with diluted ammonia before adding three drops of dimethylglyoxime, which would give a pink precipitate in the presence of nickel(II).

(III) Testing for antimony(III) was performed by adding aqueous Na_2_S upon which the negligibly water‐soluble Sb_2_S_3_ should precipitate as red solid. None of the reactions expected for positive testing was observed.

(IV) For tungsten(VI) testing, the solution was acidified with diluted H_2_SO_4_ before adding a spatula tip of zinc powder. Observed gas evolution is attributed to hydrogen that originates from the oxidation of zinc by H_2_SO_4_ and can partly reduce tungsten(VI), forming the characteristic tungsten blue.

(V) To confirm the presence of lead(II) in the PbO and PbO_2_ samples for CV, 2 mL of 0.5 m Na_2_CO_3_ solution were added. The white precipitate was centrifuged, washed with water two more times and one time with ethanol before drying in the vacuum drying oven at room temperature. The presence of PbCO_3_ in both precipitates was confirmed by PXRD.

### Control experiments for testing the influence of urea/ammonia

(I) Cu_2_O was heated at 150 °C in a mixture of [Hbet][NTf_2_] and [Hbet]Cl in a molar ratio of 1 : 2 : 2 open to the air (a) without any additives, (b) with the fourfold amount of urea or (c) 6 drops of 25 % aqueous ammonia solution. In the urea‐ and ammonia‐doped samples (b) and (c), Cu_2_O completely dissolved to form a blue solution within 10 min, while no reaction was observed for sample (a).

(II) 1 g of freshly synthesized [Hbet]Cl/4U/2.5GLY was evacuated over night with dynamic vacuum at 10^−2^ mbar at room temperature. Subsequently, Cu_2_O was added in a molar ratio of *n*_Cu_ : *n*_[Hbet]Cl_=1 : 4 in an argon‐filled glove box (MBraun; *p*(O_2_)/*p* ^0^<1 ppm, *p*(H_2_O)/*p* ^0^<1 ppm). The sealed flask was stirred at 150 °C for 4 h before the stopper was removed to allow contact to air. After initial light blue coloration, a slightly yellow solution was obtained after 4 h under argon atmosphere. Upon exposure to air, the sample rapidly turned intensely blue.

(III) 246 mg [Hbet]Cl (= mass contained in 1 g DES) was dissolved in 2 mL glycerol within 2 h at 80 °C. GeO_2_ was added in a molar ratio of *n*_Ge_ : *n*_[Hbet]Cl_=1 : 4 together with (a) no additional substances, (b) the fourfold amount of urea or (c) 8 drops of 25 % aqueous ammonia solution (added to the hot sample). At 150 °C, samples (b) and (c) formed clear solutions within 20 min or a few seconds, respectively, while no reaction was observed for sample (a).

**Powder X‐ray diffractometry**. Powder X‐ray diffraction (PXRD) was performed on two equivalent X'Pert and Empyrean diffractometers (PANalytical) equipped with a curved Ge(111) monochromator in Bragg‐Brentano geometry at room temperature using Cu−Kα_1_ radiation (*λ*=154.056 pm).

**Nuclear magnetic resonance spectroscopy**. Undissolved samples were filled in a NMR tube together with a capillary filled with DMSO‐d_6_. Measurements were performed on a Bruker Avance Neo WB 300 MHz NMR spectrometer at resonance frequencies of 300 MHz for ^1^H and 75.5 MHz for ^13^C spectra. The chemical shifts were externally referenced according to tetramethylsilane (TMS).

**Mass spectrometry**. For mass spectrometry, a 1260 infinity HPLC coupled with 6538 UHD Accurate Mass Q‐TOF LC/MS was used. The samples were dissolved in purified water and diluted 1 : 1000. A bypass instant of a chromatographic column was used to transport the samples to the mass spectrometer. For the electrospray ionization, the following parameters were used: nebulizer pressure 40 psig, fragmentor voltage 60 V, capillary voltage 3 kV, drying gas temperature 325 K and gas flow 8 L min^−1^. The measurements were performed in positive mode, only detecting cations.

**Sample preparation for electrochemistry**. Solutions of a metal oxide in [Hbet]Cl/4U/2.5GLY were prepared with a molar ratio of *n*_M_ : *n*_DES_=1 : 8 by stirring at 150 °C in an open round‐bottom flask until no solid was observable (copper, manganese, molybdenum, lead and zinc oxides) or for 4 h (cobalt, tin and vanadium oxides). Sample sizes were based on 2 g DES.

**Cyclic voltammetry**. CV measurements were performed on a Biologic VMP3 potentiostate in a three‐electrode electrochemical cell with a glassy carbon disk working electrode (diameter 3 mm), a platinum plate counter electrode and a platinum wire pseudo‐reference electrode in different ranges from −2.3 V to 0.5 V and with a scan rate of 10 mV/s at 60 °C. Prior to the measurement, the platinum electrodes were rinsed with water and ethanol and cleaned with fuzz‐free tissue, before drying in air. The working electrode was polished with 0.05 μm alumina paste on a velvet pad, rinsed with water and ethanol and dried in air.

**Electrochemical metal deposition**. Samples were prepared as described for CV. Electrodeposition experiments were performed in a three‐electrode electrochemical cell with a carbon cloth working electrode, a platinum plate as counter electrode and a platinum wire as pseudo‐reference electrode by applying different potentials for 1 h at 60 °C. Prior to the measurement, the platinum electrodes were rinsed with water and ethanol and cleaned with fuzz‐free tissue, before drying in air.

**Energy‐dispersive X‐ray spectroscopy**. For EDX measurements, the carbon cloth electrodes were fixed on the sample holder by a carbon pad. Semi‐quantitative energy‐dispersive X‐ray spectroscopy (*U*_a_=15–20 kV) was performed using a SU8020 electron microscope (Hitachi) equipped with an Oxford Silicon Drift Detector X–MaxN.

## Supporting Information Summary

The authors have cited additional references within the Supporting Information.^[67]^ The Supporting Information contains temperature‐dependent ^1^H NMR spectra of the DES, observations and PXRD data of the metal oxide dissolution experiments and EDX maps of the working electrodes after metal electrodeposition.

## Conflict of interest

The authors declare no conflict of interest.

1

## Supporting information

As a service to our authors and readers, this journal provides supporting information supplied by the authors. Such materials are peer reviewed and may be re‐organized for online delivery, but are not copy‐edited or typeset. Technical support issues arising from supporting information (other than missing files) should be addressed to the authors.

Supporting Information

## Data Availability

The data that support the findings of this study are available from the corresponding author upon reasonable request.
