# Supplementary material for: Critical Investigation of Betaine Hydrochloride‐Based Deep Eutectic Solvent for Ionometallurgical Metal Production
Source: ChemistryOpen. 2023 Aug 7;12(8):e202300114. doi: 10.1002/open.202300114 (PMC10405249; doi:10.1002/open.202300114)
Supplement: Supplementary file 1 — Supporting Information [file OPEN-12-e202300114-s001.pdf]

ChemistryOpen

Supporting Information

Critical Investigation of Betaine Hydrochloride-Based Deep Eutectic Solvent for Ionometallurgical Metal Production

Janine Richter, Tobias Pietsch, Noah Elsner, and Michael Ruck*

Table of Contents

DES stability 2

Figure S1 2

Metal oxide dissolution 2

Table S1 2

Figure S2 4

Figure S3 5

Figure S4 6

Figure S5 6

Figure S6 7

Figure S7 7

Metal electrodeposition 7

Figure S8 7

Figure S9 8

Figure S10 8

Figure S11 8

Figure S12 9

Figure S13 9

Figure S14 9

Figure S15 10

Figure S16 10

Figure S17 10

Figure S18 11

Figure S19 11

References 11

DES stability

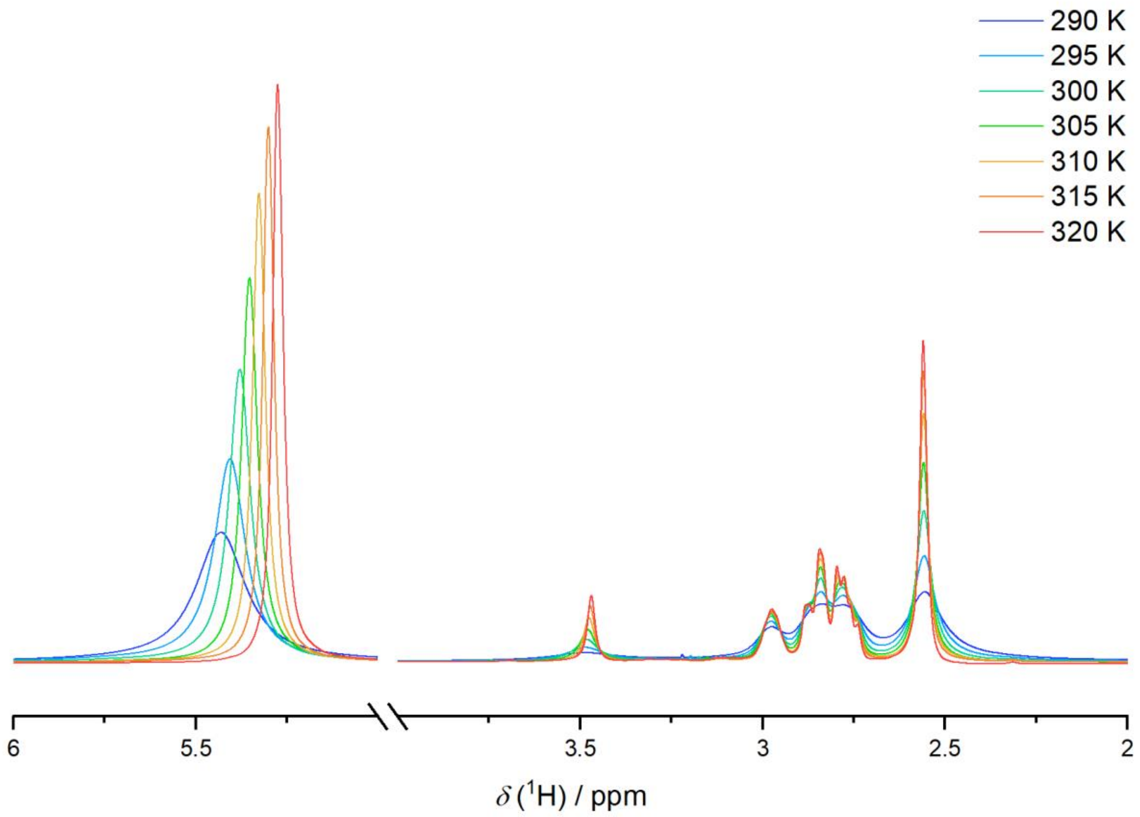

Figure S1. ^{1}H NMR spectra of [Hbet]Cl/4U/2.5GLY measured at temperatures between 290 and 320 K

Metal oxide dissolution

Table S1. Product appearance, mass fractions of the recovered solid and phases identified by PXRD of the reaction mixtures of a metal oxide and [Hbet]Cl/4U/2.5GLY. A molar ratio of n_M : n_{DES} = 1 : 4 and heating to 150 °C for a maximum of 24 h was applied. If solid residues remained, the liquid was diluted with water and removed by centrifugation before PXRD.

Metal oxide	Solubility	Observations	Recovered solid mass amount*	PXRD
Al_2O_3	negligible	white powder in brown liquid	94 %	Al_2O_3
BaO	Full	Clear, colorless solution after 5 min	-	-
Bi_2O_3	High	Fast formation of a voluminous, white powder, unstirrable brown mass after 18 h, washing gives grey powder and brown liquid	109 %	Bi_2O_3, $Bi_2O_3CO_3$
CaO	Full	Clear, colorless solution after 10 min	-	-
CoO	High	Rapid blue coloration of solution, black powder in blue solution after 24 h	25 %	CoO, Co_3O_4

Co_3O_4	High	Rapid blue coloration of solution, black powder in blue solution after 24 h	9%	Co_3O_4, CoO
Cr_2O_3	negligible	Green powder in brown liquid	84 %	Cr_2O_3
Cu_2O	Full	Dark blue solution after 45 min	-	-
CuO	Full	Dark blue solution after 2 h, blue precipitate	-	-
Fe_2O_3	negligible	Red powder in brown liquid	89 %	Fe_2O_3
FeOOH	negligible	Yellow powder in orange liquid	100 %	FeOOH
Ga_2O_3	negligible	White powder in brown liquid	84 %	Ga_2O_3
GeO_2	Full	Clear, colorless solution after 1 h	-	-
$LiCoO_2$	High	Rapid blue coloration of solution, black powder in blue solution after 24 h	0 %	$LiCoO_2$
MgO	Full	Clear, colorless solution after 1 h	-	-
MnO	Full	Clear, slightly brownish solution after 10 min	-	-
MnO_2	High/Full	85-90 % pure MnO_2: brown solution after 3 h; 99 % pure MnO_2: very small amount of black powder in dark brown liquid	0 %	MnO_2, some unidentified reflections
MoO_3	Full	Brown solution after 30 min	-	-
NiO	negligible	Pale green powder brown liquid	97 %	NiO
PbO	Full	Clear, colorless solution after 10 min	-	-
PbO_2	Full	Clear, yellow solution after 10 min	-	-
Sb_2O_3	negligible	White powder in orange liquid after 24 h	100 %	Sb_2O_3
SnO	High	White and black powder in brown liquid	98 %	SnO, unidentified reflections
SrO	Full	Clear, colorless solution after 10 min	-	-
TiO_2	negligible	White powder in brown liquid	85 %	TiO_2
V_2O_3	Low	Black powder in brown liquid after 24 h	89 %	V_2O_3, V_3O_5
VO_2	Low	Black powder in brown liquid after 24 h	76 %	VO_2, V_6O_{13}
V_2O_5	Full	Brown powder in brown liquid after 24 h	121 %	No reflection
WO_3	Low	Yellow powder in orange liquid after 24 h	54 %	WO_3
ZnO	Full	Clear, colorless solution after 5 min	-	-

* The values only serve as rough orientation as mass losses have to be considered during wet-chemical work-up.

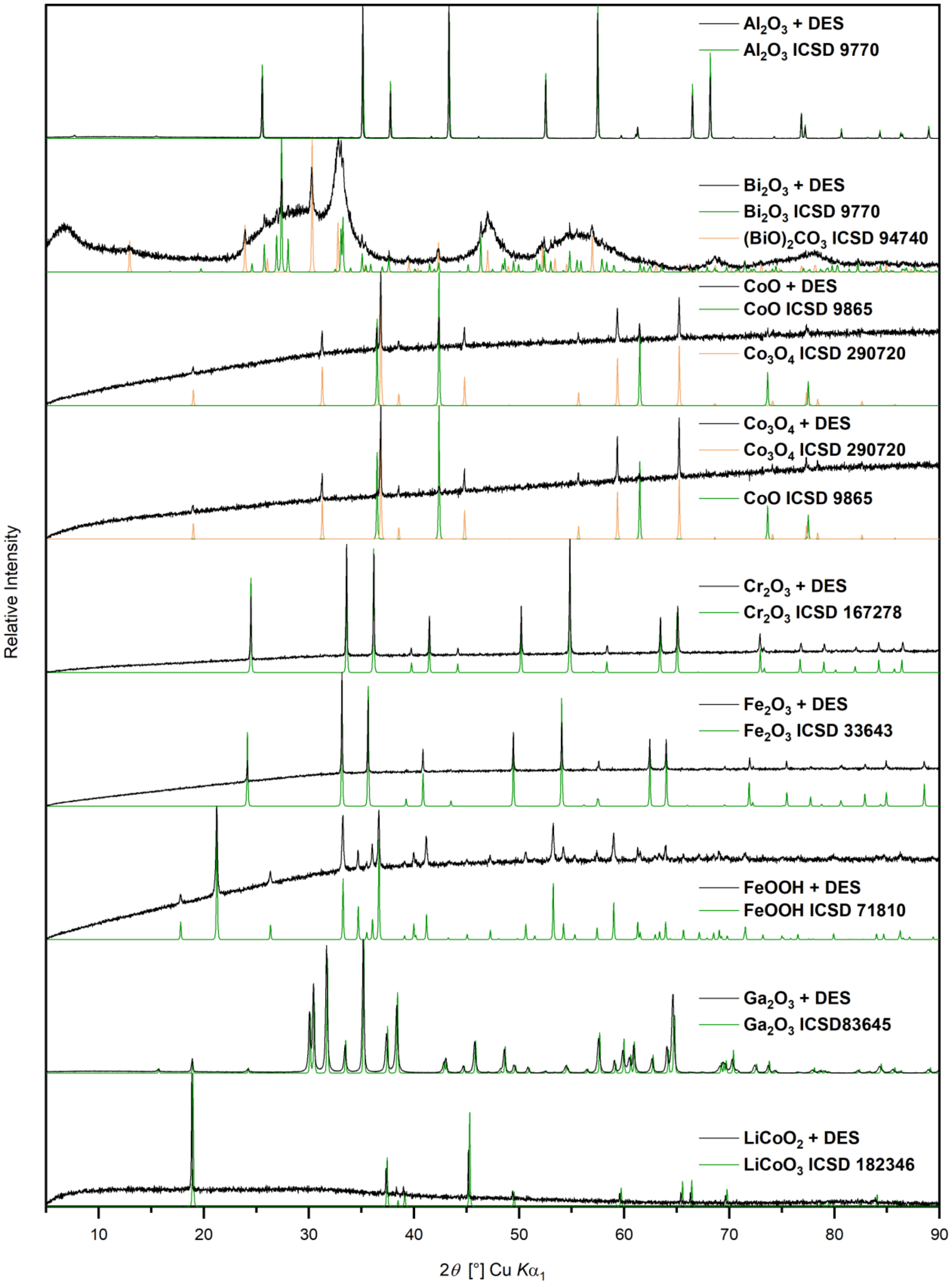

Figure S2. Experimental diffractograms of the samples Al_2O_3, Bi_2O_3, CoO, Co_3O_4, Cr_2O_3, Fe_2O_3, FeOOH, Ga_2O_3, and $LiCoO_2$ + [Hbet]Cl/4U/2.5GLY (black) in the range 5° ≤ 2θ ≤ 90° compared to the reflection patterns of the respective metal oxide if present (green) and other identified phases (orange) simulated from single crystal data.

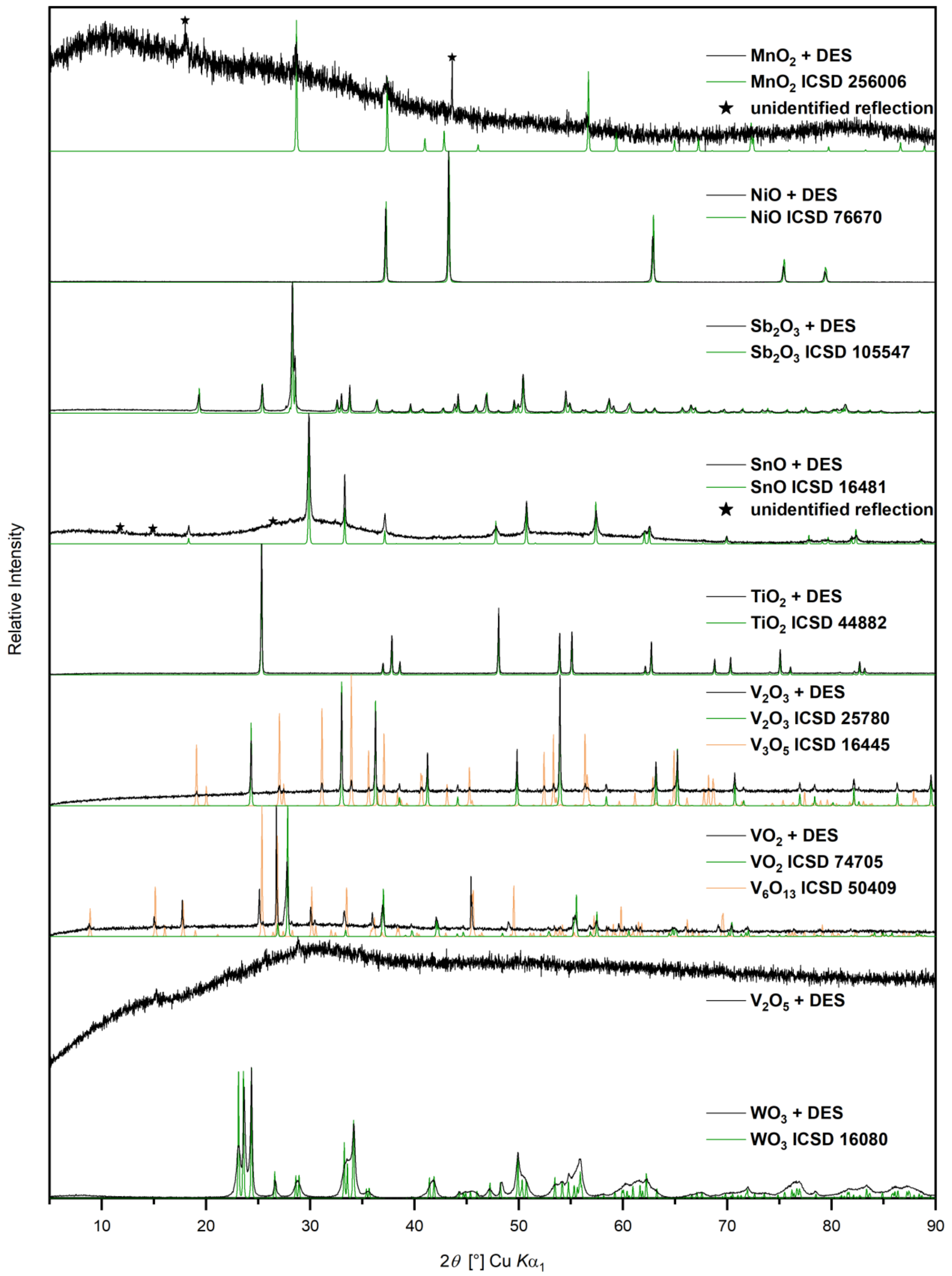

Figure S3. Experimental diffractograms of the samples MnO_2, NiO, Sb_2O_3, SnO, TiO_2, V_2O_3, VO_2, V_2O_5 and WO_3 + [Hbet]Cl/4U/2.5GLY (black) in the range 5° ≤ 2θ ≤ 90° compared to the reflection patterns of the respective metal oxide if present (green) and other identified phases (orange) simulated from single crystal data.

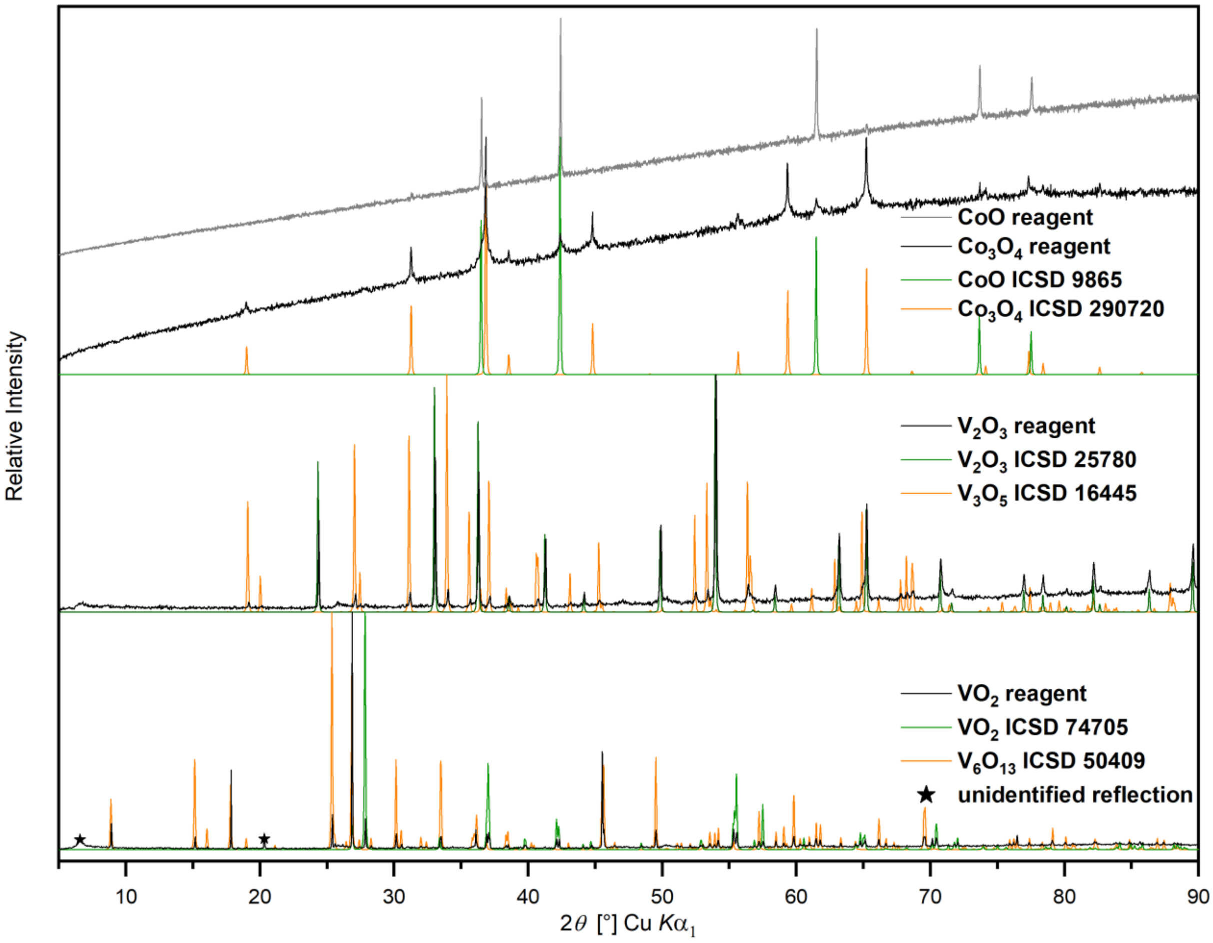

Figure S4. Measured diffractograms of the reagents CoO (grey), Co_3O_4, V_2O_3 and VO_2 (black) compared to the patterns of respective metal oxides (green and orange) calculated from single-crystal data in the range 5° ≤ 2θ ≤ 90°.

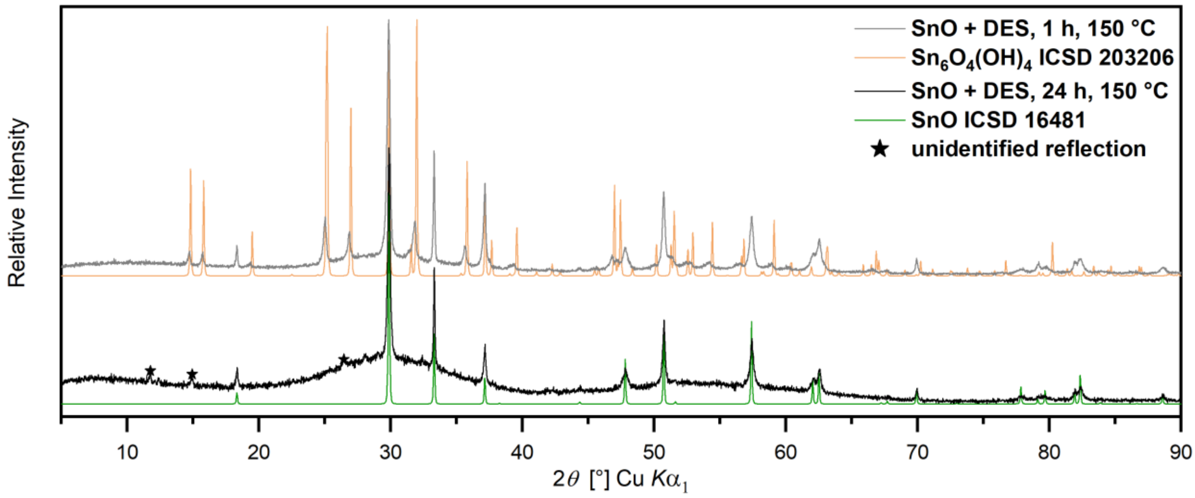

Figure S5. Diffractograms of solid residues of SnO reacted in [Hbet]Cl/4U/2.5GLY at 150 °C for 1 h (grey) or 24 h (black) compared to the diffractograms of $Sn_6O_4(OH)_4$ (orange) and SnO (green) calculated from single-crystal data in the range 5° ≤ 2θ ≤ 90°.

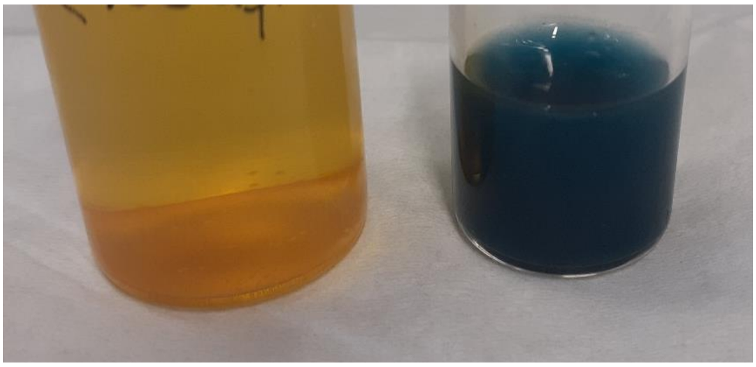

Figure S6. Evidence for the presence of dissolved tungsten(VI) after WO_3 was stirred in [Hbet]Cl/4U/2.5GLY for 24 h at 150 °C. The left solution shows the solution after dilution with water and separation from solid residual. The color changes in the vial on the right after addition of diluted sulfuric acid and zinc powder.

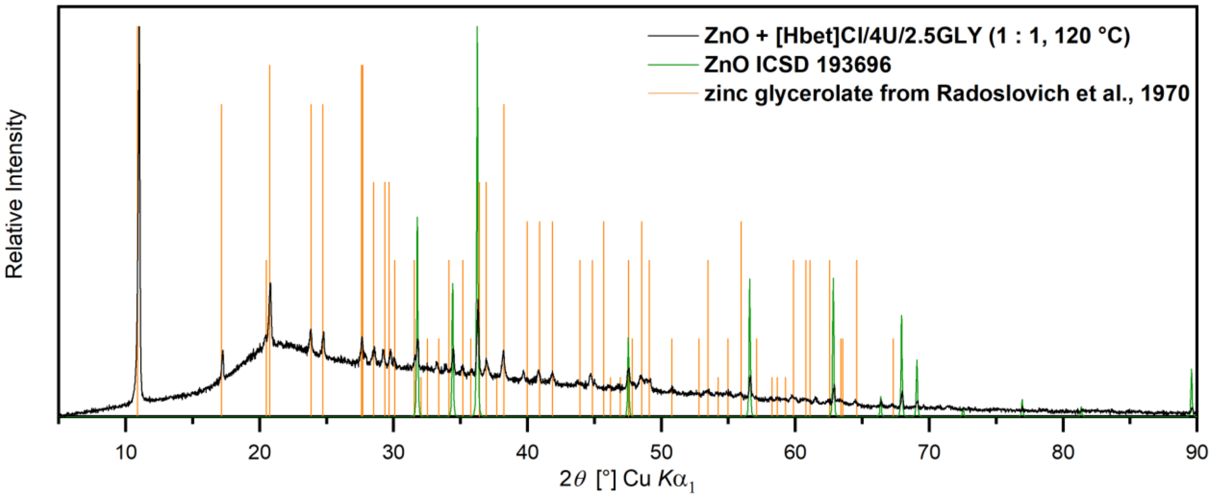

Figure S7. Measured diffractogram of ZnO reacted in [Hbet]Cl/4U/2.5GLY (n_{ZnO} : n_{DES} = 1 : 1) at 120 °C (black) compared to the patterns of ZnO (green) calculated from single-crystal data in the range 5° ≤ 2θ ≤ 90° and zinc glyerolate. The reflection positions and intensities of zinc glycerolate (orange) were obtained from [1].

Metal electrodeposition

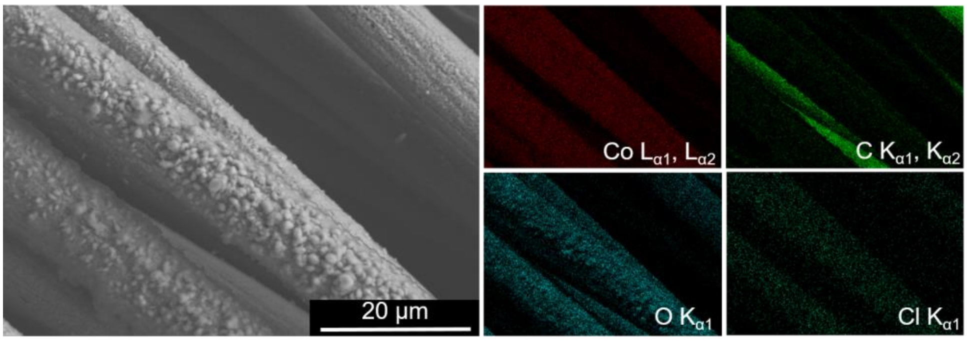

Figure S8. SEM image and EDX maps of a section of the carbon cloth electrode immersed into the solution of CoO in [Hbet]Cl/4U/2.5GLY after electrodeposition at 60 °C and −1.7 V for 1 h and rinsing with water and ethanol. Detected chlorine is attributed to an incomplete removal of the DES, oxygen to corrosion due to storage on air.

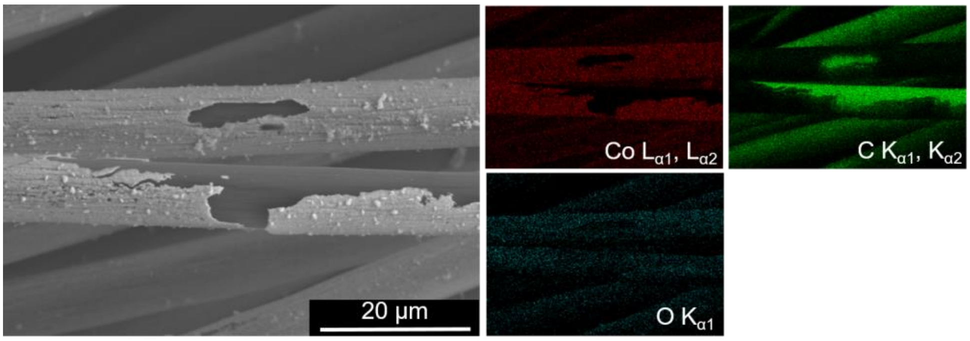

Figure S9. SEM image and EDX maps of a section of the carbon cloth electrode immersed into the solution of Co_3O_4 in [Hbet]Cl/4U/2.5GLY after electrodeposition at 60 °C and −1.7 V for 1 h and rinsing with water and ethanol. Detected oxygen is attributed to corrosion due to storage on air.

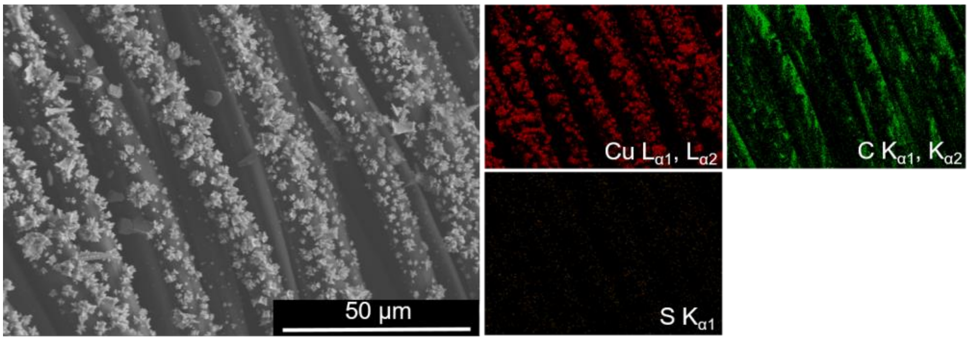

Figure S10. SEM image and EDX maps of a section of the carbon cloth electrode immersed into the solution of Cu_2O in [Hbet]Cl/4U/2.5GLY after electrodeposition at 60 °C and −1.0 V for 1 h and rinsing with water and ethanol. Detected sulfur is attributed to corrosion due to storage on air.

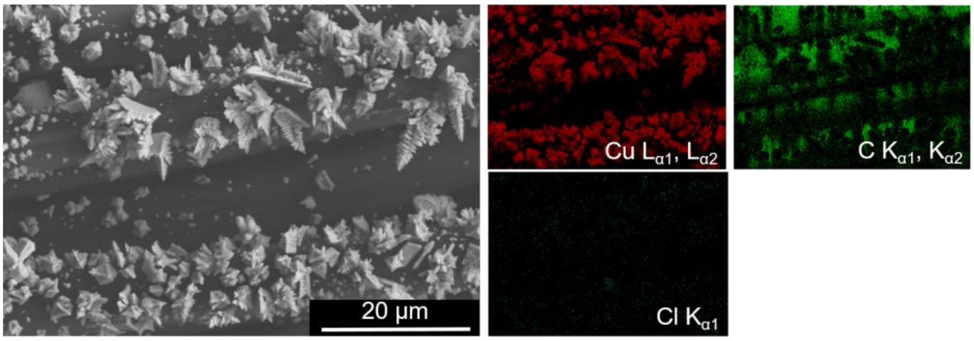

Figure S11. SEM image and EDX maps of a section of the carbon cloth electrode immersed into the solution of CuO in [Hbet]Cl/4U/2.5GLY after electrodeposition at 60 °C and −1.0 V for 1 h and rinsing with water and ethanol. Detected chlorine is attributed to an incomplete removal of the DES.

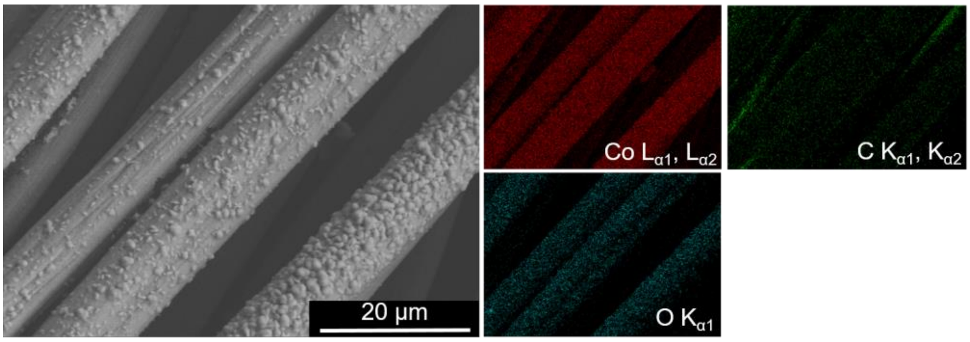

Figure S12. SEM image and EDX maps of a section of the carbon cloth electrode immersed into the solution of $LiCoO_2$ in [Hbet]Cl/4U/2.5GLY after electrodeposition at 60 °C and −1.7 V for 1 h and rinsing with water and ethanol. Detected oxygen is attributed to corrosion due to storage on air.

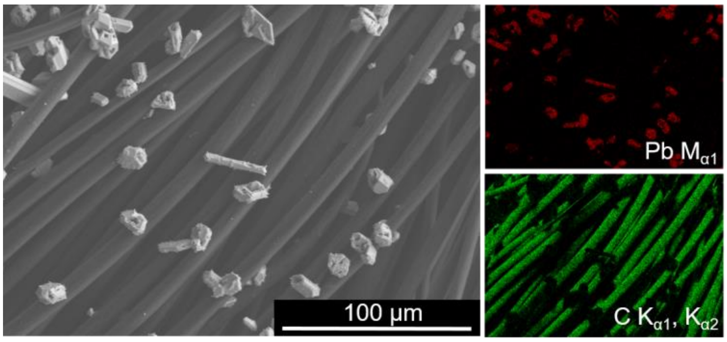

Figure S13. SEM image and EDX maps of a section of the carbon cloth electrode immersed into the solution of PbO in [Hbet]Cl/4U/2.5GLY after electrodeposition at 60 °C and −0.8 V for 1 h and rinsing with water and ethanol.

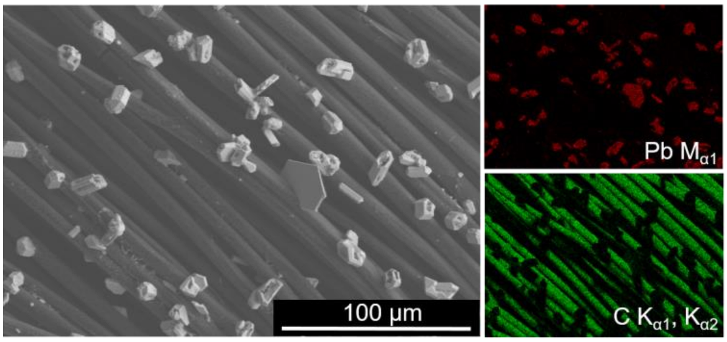

Figure S14. SEM image and EDX maps of a section of the carbon cloth electrode immersed into the solution of PbO_2 in [Hbet]Cl/4U/2.5GLY after electrodeposition at 60 °C and −0.8 V for 1 h and rinsing with water and ethanol.

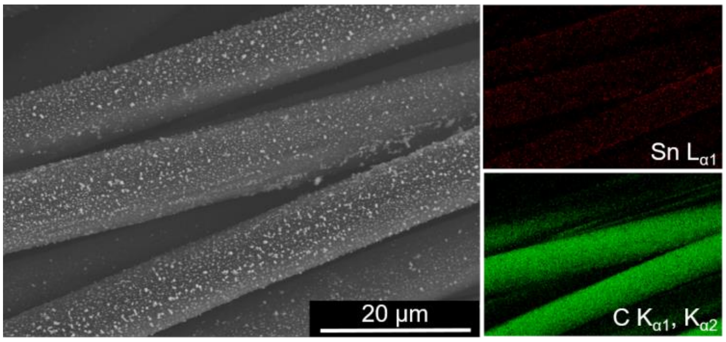

Figure S15. SEM image and EDX maps of a section of the carbon cloth electrode immersed into the solution of SnO in [Hbet]Cl/4U/2.5GLY after electrodeposition at 60 °C and −1.5 V for 1 h and rinsing with water and ethanol.

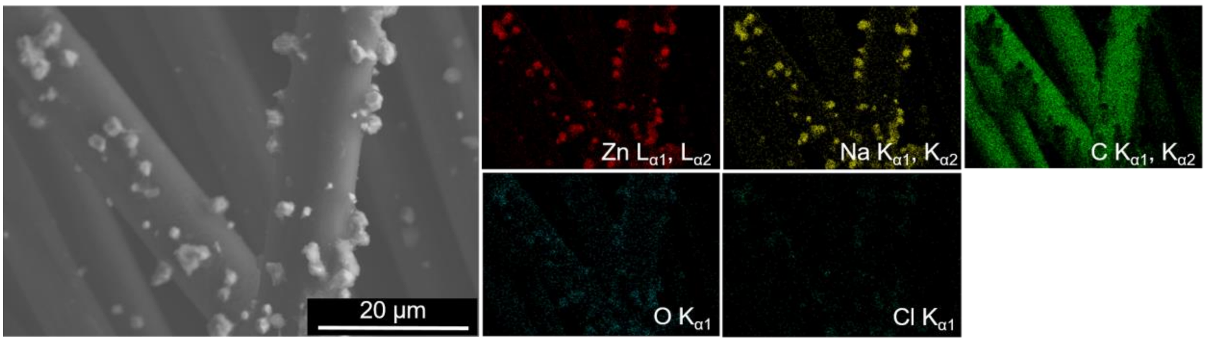

Figure S16. SEM image and EDX maps of a section of the carbon cloth electrode immersed into the solution of ZnO in [Hbet]Cl/4U/2.5GLY after electrodeposition at 60 °C and −1.5 V for 1 h and rinsing with water and ethanol. Detected chlorine is attributed to an incomplete removal of the DES, oxygen to corrosion due to storage on air. Sodium is detected because zinc and sodium EDX signals overlap.

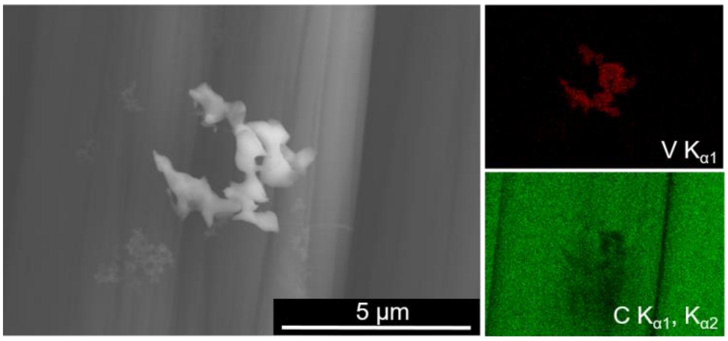

Figure S17. SEM image and EDX maps of a section of the carbon cloth electrode immersed into the solution of V_2O_3 in [Hbet]Cl/4U/2.5GLY after electrodeposition at 60 °C and −2.3 V for 1 h and rinsing with water and ethanol.

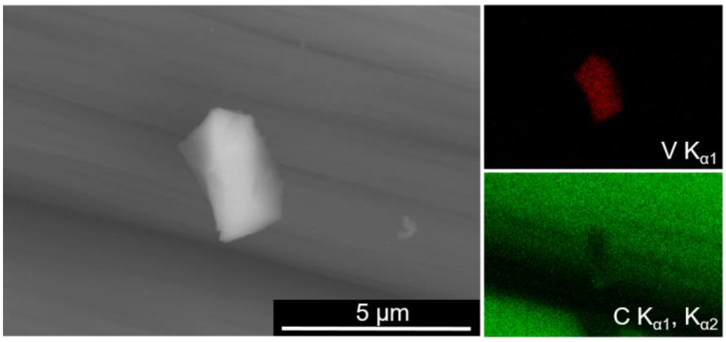

Figure S18. SEM image and EDX maps of a section of the carbon cloth electrode immersed into the solution of VO_2 in [Hbet]Cl/4U/2.5GLY after electrodeposition at 60 °C and −2.3 V for 1 h and rinsing with water and ethanol.

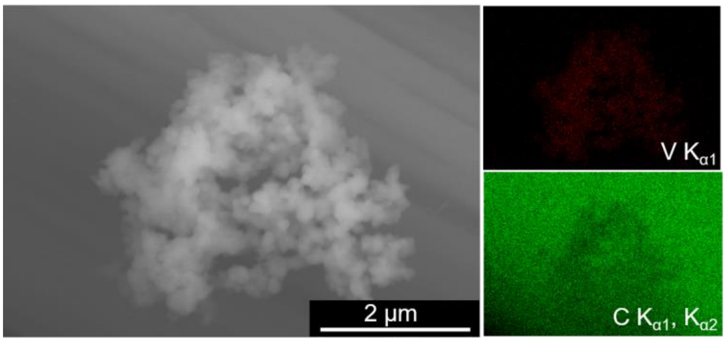

Figure S19. SEM image and EDX maps of a section of the carbon cloth electrode immersed into the solution of V_2O_5 in [Hbet]Cl/4U/2.5GLY after electrodeposition at 60 °C and −2.3 V for 1 h and rinsing with water and ethanol.

References

[1] E. Radoslovich, M. Raupach, P. Slade, R. Taylor, *Aust. J. Chem.* **1970**, *23*, 1963.
